# Supplementary material for: Nasogastric tube in mechanical ventilated patients: ETCO2 and pH measuring to confirm correct placement. A pilot study
Source: PLoS One. 2022 Jun 2;17(6):e0269024. doi: 10.1371/journal.pone.0269024 (PMC9162373; doi:10.1371/journal.pone.0269024)
Supplement: S1 Appendix — (DOCX) [file pone.0269024.s001.docx]

**Tracheal etCO2 level and gastric pH level measurements during the correct naso-gastric tube placement in unconscious patients. A physiological, prospective, observational study**

Study Type: Other Clinical Trial according to ClinO, Chapter 4

Risk Categorisation: Category A. Non-interventional study including research related

procedures with minimal risks and burdens

Protocol ID CLM_ICU_001

Principal Investigator Dr. med. Samuele Ceruti

Clinica Luganese Moncucco

Via Moncucco 10a, 6900 - Lugano

Phone.: +41(0) 91 960.81.11

Email: samuele.ceruti@moncucco.ch

Investigated Intervention: Tracheal etCO2 level and gastric pH level measurements during the correct naso-gastric tube placement in unconscious patients. A physiological, prospective, observational study

Version and Date: Version 3.0, 11 Feb 2020

**Protocol Signature Form**

| Study Title | Tracheal etCO2 level and gastric pH level measurements during the correct naso-gastric tube placement in unconscious patients. A physiological, prospective, observational study |
| --- | --- |
| Study ID | CLM_ICU_001 |

The Project Leader has approved the protocol version ***[3.0 (dated 11 Feb 2020)]****,* and confirms hereby to conduct the project according to the protocol, the Swiss legal requirements [1, 2], current version of the World Medical Association Declaration of Helsinki [3] and the principles of Good Clinical Practice (GCP).

**Principal Investigator:**

Name: Dr.med. Samuele Ceruti

Place/Date: Signature:

**Local Principal Investigator at study site:**

I have read and understood this study protocol and agree to conduct the study as set out in this study protocol, the current version of the World Medical Association Declaration of Helsinki, ICH-GCP guidelines and the local legally applicable requirements.

**Site**:

Ospedale Regionale di Bellinzona e Valli, Bellinzona

**Principal Investigator**:

Name*:* PD Dr.med. Andrea Saporito

Place/Date: Signature:

# Table of Contents

Table of Contents 3

GLOSSARY OF ABBREVIATIONS 4

1 STUDY SYNOPSIS 5

2 ADMINISTRATIVE STRUCTURE 7

2.1 Project Leader 7

2.2 Principal Investigators 7

2.3 Co-Investigators 7

2.4 Sub Investigators 8

2.5 Clinical Study Coordinators 8

3 BACKGROUND and rationale 9

3.1 Background 9

3.2 Project rationale and significance 10

4 Study OBJECTIVES and Design 10

4.1 Primary and secondary endpoints 10

4.2 Study design 11

4.3 Study intervention 11

5 Study POPULATION and Study procedures 13

5.1 Inclusion and exclusion criteria, justification of study population 13

5.2 Recruitment, screening and informed consent procedure 13

5.3 Withdrawal and discontinuation 13

5.4 Project Schedule 14

5.5 Study procedures 14

6 STATISTICS AND METHODOLOGY 14

6.1 Determination of sample size 14

6.2. Statistical analysis plan 14

7 Regulatory Aspects AND SAFETY 15

7.1 Local regulations / Declaration of Helsinki 15

7.2 Serious Events 15

7.3 Amendments 15

7.4 End of the project 15

7.5 Insurance 15

8 FURTHER Aspects 16

8.1 Overall ethical considerations 16

8.2 Risk-benefit assessment 16

9 Quality CONTROL AND Data protection 16

9.1 Quality measures 16

9.2 Data recording and source data 16

9.3 Confidentiality and coding 17

9.4 Retention and destruction of study data and biological material 17

10. Funding / Publication / declaration of Interest 17

11. REFERENCES 17

Appendix 1: Tab. 1 21

GLOSSARY OF ABBREVIATIONS

*AE Adverse Event*

*ASR/DSUR Annual Safety Repot / Development Safety Report*

*BASEC Business Administration System for Ethical Committees*

*ClinO Ordinance on Clinical Trials in Human Research*

*COPD Chronic Obstructive Pulmonary Disease*

*CRF Case Report Form*

*CTCAE Common Terminology Criteria for Adverse Events*

*EC Ethics Committee*

*eCRF electronic Case Report Form*

*ENT Ears, Nose and Throat*

*etCO2 end-tidal CO2*

*FADP Federal Act on Data Protection*

*FOPH Federal Office of Public Health*

*GCP Good Clinical Practice*

*HRA Human Research Act*

*ICH International Conference on Harmonisation*

*ICU Intensive Care Unit*

*ITT* I*ntention-To-Treat*

*NGT Naso Gastric Tube*

*PP* *Per-Protocol*

*PPIs Proton Pump Inhibitors*

*SAE Serious Adverse Event*

# STUDY SYNOPSIS

| **Project Leader** | Dr. Med. Samuele Ceruti  Clinica Luganese Moncucco  Via Moncucco 10a, 6900 - Lugano  phone.: +41 (0)91 960.81.11  email: samuele.ceruti@moncucco.ch |
| --- | --- |
| **Study Title** | Tracheal etCO2 level and gastric pH level measurements as markers to confirm during the correct naso-gastric tube placement in unconscious patients. A physiological, prospective, observational study |
| **Study ID** | CLM_ICU_001 |
| **Protocol Version and Date** | Version 2, 31 Dec 2019 |
| **Risk Categorisation** | Risk category A according to HRO Art. 7 |
| **Study Design** | Prospective, observational study |
| **Project Design** | A multisite, prospective, observational, study in patients who required a NGT placement.  Group A: etCO2 measurement will be collected 1. after intubation, when the tube is inserted into the endotracheal tube, before proceeding with the aspiration of secretions and 2. once the NGT has been inserted, by a probe located at the end of the tube  Group B: pH measurement will be collected at the end of the procedure, once the NGT is inserted, at 1.a distance of 25 cm from the mouth (esophageal site) and at 2. a distance of 40 cm (gastric site), aspirating the gastric contents and measuring on specific litmus paper.  Group A will be done entirely in the Clinica Luganese Moncucco while group B in the Regional Hospital of Bellinzona. |
| **Background and Rationale** | The laying of a naso-gastric tube (NGT) is an extremely common procedure performed in intensive medicine; although the standard NGT insertion is made at the patient's bed, this procedure is not free of risk. In this setting, can occur serious complications, especially in the sedated, intubated and curarized patient, which does not present the cough reflex. Some studies show an incidence of complications in the NGT pose in around 1% of total insertions, but these complications are associated to a high morbidity, leading to an increase of hospital stay, global mortality and health costs.  Numerous methodologies have been evaluated to recognize the correct positioning of NGT at the gastric level, including different clinical techniques (such as gastric auscultation, aspiration of the NGT), ultrasound techniques, etc. Actually, the diagnostic gold standard is the thoraco-abdominal anterior-posterior radiography, which is considered the only non-invasive method capable to confirm the correct pose of the NGT at intra-diaphragmatic level. This method, however, even if it is non-invasive, requires the use of ionizing radiation (4 uSv for radiography) which could be repeated multiple time for the same patient; NGT may need to be repositioned several times during the same hospital stay, increasing patient exposure to ionizing radiation and, potentially, also the health workers exposure,.  Furthermore, in sedated and intubated patients, the detection cassette is placed under the back. In patients with hemodynamic or respiratory instability, the mobilization required for the detection cassette placement, appears to be a further risk to which the patient could be exposed |
| **Objectives** | To identify into the group A the threshold value between tracheal and esophageal etCO2. To identify into the group B the theshold value between gastric and esophageal pH. |
| **Endpoints** | Group A:  - the aim is to find a threshold value of etCO2 collected when the NGT is well positioned in trachea;  Group B  - the aim is to find a threshold value of pH collected when the NGT is positioned in the esophagus and in the stomach  Secondary endpoints:   - Group A: identify patients suffering from chronic obstructive pulmonary disease (COPD) and perform a sub-analysis to identify the threshold value; - Group B: identify patients taking proton pump inhibitors (PPIs) and perform a sub-anaysis to identify the threshold value in case of an already diagnosed gastro-esophageal reflux disease. |
| **Inclusion- / Exclusion Criteria** | Inclusion criteria:  • Adult patients (> 18 years)  • Patients male and female  • Patients intubated by oro- or naso-tracheal way  • Post-induction curarized patients  • Fasting patients (from at least 6 hours)  Exclusion criteria:  • Patient refusal  • Patients with known bleeding diathesis / ongoing bleeding  • Patients at risk of bleeding (defined as thrombocytes <50 G/l, INR> 2.5, aPTT> 70 sec)  • Patients with traumatic brain injury / Polytrauma  • Patients with esophagus-tracheal fistulas or malformations of the ENT sphere  • Patients with current or previous radiotherapy of the ENT sphere  • Patients unable to give their informed consent due to language barriers  • Women who are pregnant |
| **Procedures and Assessments** | Clinical data will be collected at the NGT positioning |
| **Number of Participants with Rationale** | The study will include a total number of 75 patients (group A: 40 pts, group B: 35 pts) |
| **Study Duration and Schedule** | Planned 01/2020 of First-Participant-In  Planned 01/2021 of Last-Participant-Out |
| **Statistical Considerations** | Sample size: in order to have a significant difference between tracheal and esophageal groups, with a power of 90% and a significance level of 0.01 (one-tailed z test), and anticipating a 10% in the registration failure rate, we plan to enroll 40 patients for group A. In order to have a significant difference between esophageal and gastric pH value, with a power of 90% and a significance level of 0.01 (one-tailed z test), and anticipating a 10% of drop off rate, we plan to enroll 35 patients for group B.  Primary analysis: The adherence to the protocol is checked for each enrolled patient during the study. We assess and report total adherence rate to our protocol in order to analyse data on the intention-to-treat (ITT) and on the per-protocol (PP) population. We test the hypothesis of difference in the proportion of etCO2 level into the tracheal group and into the esophageal group (measurements collect in patients enrolled in group A), with the ITT. We execute a z-test for comparison of the two proportions. The null hypothesis of no difference between the two proportions is not accept if the p-value is 0.01. Similarly, in group B patients is test the hypothesis of difference in the proportion of pH level into the esophageal group and into the gastric group, with the ITT. We carry out a z-test for comparison of the two proportions. The null hypothesis of no difference between the two proportions is not accept if the p-value is 0.01. |
| **Risk / Benefit Assessment** | This study consists in parameters collection in patients who required a NGT positioning. Any potential clinical risk is related only to the standard procedure of NGT placing.  There will be no immediate benefits to the enrolled patients. Future patients who required a NGT positioning could benefit due to the potential of using threshold values as predictors of correct NGT placing, offering better treatment approach which not required thoraco-abdominal anterior-posterior radiography as diagnostic gold standard. |

# ADMINISTRATIVE STRUCTURE

## Project Leader

The name and contact details of the Project Leader of the study are below:

*Dr. Med. Samuele Ceruti*

Service of Intensive care unit and emergency care

Clinica Luganese Moncucco

Via Moncucco 10, 6900 - Lugano

phone.: +41 (0)91 960.81.08

email: samuele.ceruti@moncucco.ch

Dr. med. Samuele Ceruti is responsible for the study design, protocol development, overall study conduction and final results interpretation and reporting.

## Principal Investigators

This is a multisite study that will be conducted at the Clinica Luganese Moncucco of Lugano and at the Ospedale Regionale di Bellinzona e Valli, Bellinzona.

The Principal Investigator at the Clinica Luganese Moncucco of Lugano is:

Dr. med. Samuele Ceruti (see contact details under Section 2.1).

The Principal Investigator at the Ospedale Regionale di Bellinzona e Valli of Bellinzona is:

*PD Dr. med. Andrea Saporito*

Vice Head of the Anesthesiology Division

Ospedale Regionale di Bellinzona e Valli

Bellinzona (Switzerland)

phone: +41 (0)91 811 89 78

email: [andrea.saporito@eoc.ch](mailto:andrea.saporito@eoc.ch)

## Co-Investigators

*Dr.med. Romano Mauri*

Head of Service of Intensive care unit and emergency care

Clinica Luganese Moncucco

Phone: +41 (0)91 960.80.90

email: [romano.mauri@moncucco.ch](mailto:romano.mauri@moncucco.ch)

## Sub Investigators

*Dr. med. Michele Musiari*

Anesthesiology Division

Ospedale Regionale di Bellinzona e Valli

Bellinzona (Switzerland)

phone: +41 (0)91 811 89 78

email: [michele.musiari@eoc.ch](mailto:michele.musiari@eoc.ch)

*Dr. med. Marco Spagnoletti*

Service of Intensive care unit and emergency care

Clinica Luganese Moncucco

phone.: +41 (0)91 960 85 23

email: marco.spagnoletti@moncucco.ch

*Dr. med. Pietro Greco*

Service of Intensive care unit and emergency care

Clinica Luganese Moncucco

phone.: +41 (0)91 960 85 36

email: pietro.greco@moncucco.ch

## Clinical Study Coordinators

*PhD Maira Biggiogero*

Clinical Research Unit

Clinica Luganese Moncucco

Via Moncucco, 10 6900 Lugano

phone : +41 (0)91 960 86 66

email : maira.biggiogero@moncucco.ch

*Dr. med. Giorgia Lo Presti*

Clinical Research Unit

Clinica Luganese Moncucco

Via Moncucco, 10 6900 Lugano

phone : +41 (0)91 960 87 95

email : [giorgia.lopresti@moncucco.ch](mailto:giorgia.lopresti@moncucco.ch)

# BACKGROUND and rationale

## 3.1 Background

The laying of a naso-gastric tube (NGT) is an extremely common procedure performed in intensive medicine; although the standard NGT insertion is performed at the patient's bed, this procedure is not free of risk. In this setting, serious complications can occur, especially in the sedated, intubated and curarized patient, which does not present the cough reflex; for example, complications in the placement of NGT in the lungs could be consequent potential pneumothorax, pneumonia *ab ingestis*, development of a bronco-pleural fistula, alveolar hemorrhages, etc. Some studies show an incidence of complications in the NGT pose in around 1% of total insertions, which are associated to a high morbidity, leading to an increase of hospital stay, global mortality and health costs (1-8, 10-11, 13-14, 16, 19, 21-24, 36, 38, 45, 53-56, 63-65, 67-69).

Numerous methodologies have been evaluated to recognize the correct positioning of NGT at the gastric level, including different clinical techniques (such as gastric auscultation, fluid from the NGT), ultrasound techniques, etc (7, 15, 18, 39-40, 43, 62). Actually, the diagnostic gold standard is the thoraco-abdominal anterior-posterior radiography, which is considered the only non-invasive method capable of confirming the correct NGT pose at intra-diaphragmatic level. However, this non-invasive method requires the use of ionizing radiation (4 uSv for radiography) which could be repeated multiple time for the same patient. As NGT may need to be repositioned several times during the same hospital stay, will be increase ionizing radiation patient exposure. Moreover, there could be an increase to ionizing radiation exposure for health workers too.

Furthermore, in sedated and intubated patients, the detection cassette is placed under the back. As some patients is hemodynamic or respiratory unstable, the required mobilization in order to place the detection cassette in situ, appears to be a further risk.

Many studies has been performed in this field, with the aim to reduce the need of conventional radiography. Chun et al showed how the positioning of the NGT in patients anesthetized, paralyzed and intubated is often difficult due to absence in patient's cooperation; the greater resistance in the NGT passage is localized in the pyriform sinus and in the arytenoid cartilages, which could cause the rolling of the NGT around the oropharynx as already published by Ozer et al. The studied methods resulted ineffective. In the study of Prasad et al the distal tip of the NGT should be lubricated with 2% lidocaine, just as the proximal lumen is filled for 0.5 - 1 cm. When the tip passes the gastresophageal junction and enters into the stomach, there is the formation of a bubble at the distal level due to the presence of gas, the bubble does not form if the NGT is positioned wrongly, or it is repeatedly formed if the NGT is in the trachea. This study, in the current state, does not seem promising(15-16).

Nguyen et al present a case report where it is shown that the use of two-dimensional ultrasound confirms the positioning of the NGT both through the soft tissues of the neck and through the epigastric window; the use of ultrasound at the patient's bed is suggested as a rapid, new and alternative method compared to the radiological "gold standard"(47). Vigneau et al used ultrasound in 35 ICU procedures in adult patients to confirm NGT correct positioning. They demonstrate that the use of ultrasound is faster than radiography and it is sensitive enough confirming 97% of the correct NGT positioning (66). Kim et al published a clinical study designed to compare the effectiveness and limitations of conventional auscultation, pH analysis, radiography and ultrasound; the study Ultrasounds presented a low sensitivity (86%) but high specificity (67%) with a 97% PPV compared to its current gold standard, the chest X-ray; the ultrasound failed to verify gastric positioning in 6 patients (32).

Fernandez et al published a review of diagnostic studies to test pH of aspirate fluids using a litmus paper; in this way they evaluated if the NGT has been placed into the stomach. It has been noted that the color variation could be reduced based on the gastric pH, thus the litmus paper is not considered as sufficiently sensitive (17). A recent clinical trial by Gilbertson et al identified the cut off pH<5.5 to distinguish between the correct positioning in the stomach (20). A study was published by Bercik et al and by Tobin et al in which it was planned to use a magnets to locate and assist the positioning of the SNG (4, 62).

The study documents the accuracy and sensitivity of using a permanent magnet and an external computer to confirm the placement of the NGT in healthy patients. It is an expensive procedure that could not be applied in patients that perform MRI.

It is important to consider that actually the ultrasound methodology results as the most effective procedure, but it presents many limits, between them it requires a specific know how and an ultrasound scanner always available and finally it has a low sensitivity.The medical practice, according to the literature, takes into consideration the following values: etCO2 trachea mean value is 40 mmHg (range 35-45 mmHg) and etCO2 esophagus <25mmHg; pH stomach mean value 3.8 (range 3.2-4.4) and pH esophagus 8.5 (range 6-9.5).

## 3.2 Project rationale and significance

Through the use of methods already adopted in clinical field, we want to collect the values of the etCO2 from the trachea and the pH when the NGT is well positioned in stomach. . There are two methods, already present at clinical level but that need to be implemented: the capnometer and the pH-meter.

The use of the graphic capnometer (capnography) has already been demonstrated to be useful for detecting the positioning of the NGT at the tracheal level (7, 33, 61, 27, 18), even if today there are no studies able to show what is the "threshold level" of the detected CO2 to say with high specificity that NGT is certainly found in the airways (25-27, 31, 33-35, 37, 44, 46, 58-59, 61).

Furthermore, the use of the pH meter has already been remarkably demonstrated to be useful in analyzing the different pH levels in the proximal gastro-enteric tract, with differences between the stomach and the esophagus, even if there are no studies in this sense to identify the "threshold level" below which the pH certainly appears to be a gastric and non-esophageal pH (41-43, 50, 52, 60).

# Study OBJECTIVES and Design

## 4.1 Primary and secondary endpoints

The primary endpoint is to identify the threshold value in the distribution and in the frequency of etCO2 and pH values. The pH values will be compared with the performed X .

The threshold values for etCO2 and for pH will be determined in two groups: group A, with the aim to find a threshold value of etCO2 above which we can be sure that aspiration probe is in the trachea and group B, with the aim to find a threshold value of pH below which we can be certain that NGT is in the stomach.

Further secondary results are expected:

- for patients in group A it will be necessary to perform a sub-analysis for the threshold value in the group of patients suffering from COPD;

- for patients in group B, a sub-group analysis will be performed for the threshold value for patients taking proton pump inhibitors (PPIs), and / or in the presence of an already diagnosed gastro-esophageal reflux disease.

## 4.2 Study design

This is a prospective, multisite, observational, physiological study, which aims to identify two groups of values ​​regarding the measured etCO2 in the trachea and esophagus (group A group) and regarding the measured pH in the esophagus and stomach (group group B).

This study aims to be a study, taking advantage of two methods already used in clinical daily life (such as capnometry and pH measurement with litmus paper) to identify "reference values".

The study will involve two sites located in the Canton Ticino (Switzerland): the Regional Hospital of Bellinzonaand Clinica Luganese Moncucco.

Group A will be done entirely in the Clinica Luganese Moncucco while group B in the Regional Hospital of Bellinzona. In this way, the staff training will be more easily practicable. Possible bias will be avoid as the measurement in each centers will be perfomed with more than one device. The devices are calibrated by external facilities according to the international standards.

Moreover, all the staff will be involved measurement collection as the survey is not operator-based.

Inclusion and exclusion criteria will be the same for Group A and Group B patients.

This study will not interfere with the patient’s routine treatment and there will be no deviation from the standard of care. All the procedures of parameters survey will take place just once, during patient access to the ICU.

Upon study inclusion, all the parameters will be collected during the standard procedures of NGT positioning (see next paragraph for further details).

## 4.3 Study intervention

Group A procedure:

Patients needing the installation of a NGT at the Clinica Luganese Moncucco are consecutively enrolled and selected according to the inclusion and exclusion criteria listed below. The enrolled patients, after receiving adequately protocol study explanation and signed the informed consent, are sedated and intubated according to the clinical indications and the local protocols, independently from the ongoing study.

Anaesthesia is performed by a professional anaesthesiologist and an anaesthetist nurse, using standardized procedures according to local routine protocols. Induction and intubation therefore take place independently of the ongoing clinical study, independent from both indications for intubation, the choice of drugs and intubation material. At the end of the procedure, as usual, the secretions are aspirated with a suction tube of 18 Fr of 6mmdiameter. When the tube is inserted into the endotracheal tube, before proceeding with the aspiration of the secretions, a capnometer is attached to its outer end (picture A), measuring the etCO2 value for 10-15 seconds. At the end of the measurement, we proceed with the aspiration of the secretions as usual.


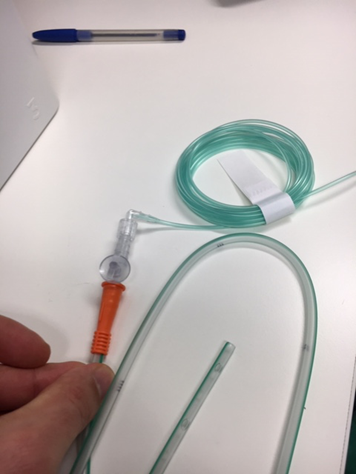
Pic. A

We then proceed with the laying of an NGT according to local protocols. Also in this case, once the NGT has been inserted, the etCO2 is measured at the end of the probe (pic A) for 10-15 seconds and its value is recorded in the CRF. At the end of the measurement, the capnometer can be detached, as a standard procedure, and the NGT can be used as usual.

At the end of the procedure, therefore, for each patient, two values ​​of etCO2 are acquired which will allow to obtain two "populations of values" of the etCO2: the values recorded ​​at the endotracheal level and the one recorded at the esophageal level. Through statistical analysis, we will find the "threshold value" above which the value of etCO2 found is certain for a location in the trachea. The expected mean values of etCO2, according to the literature, are 40 mmHg in the trachea (range 35-45 mmHg) and <25mmHg in the esophagus.

Group B procedure:

The group B enrolment will start at the Regional Hospital of Bellinzona. The enrolled patients are sedated and intubated according to the clinical indications and the local protocols, independently from the ongoing study.

Anaesthesia is performed by a professional anaesthesiologist and an anaesthetist nurse, using standardized procedures according to local routine protocols. At the end of the procedure, once the NGT is inserted, the pH is measured by aspirating the gastric contents and measuring on specific litmus paper the pH values, both at a distance of 25 cm from the mouth (esophageal site) and at a distance of 40 cm (gastric site).

At the end of the procedure, for each patient two values ​​of pH are acquired which will allow to obtain two pH "value populations": a value at esophageal level and a value at the gastric level. Through statistical analysis (see Section 6), we will try to find the "threshold value" below which the pH is suggestive for a localization in the gastric area.

The expected mean values of pH are 3.8 in the stomach (range 3.2-4.4) and 8.5 in the esophagus (range 6-9.5).

# Study POPULATION and Study procedures

## 5.1 Inclusion and exclusion criteria, justification of study population

We estimated to include 40 patients in group A (Clinica Luganese Moncucco) and 35 patients in group B (Regional Hospital of Bellinzona) for a total of 75 patients (see Section 6.1).

Inclusion criteria:

• patients need a NGT positioning;

• adult patients (> 18 years);

• patients male and female;

• patients intubated by oro- or naso-tracheal way;

• post-induction curarized patients;

• fasting patients (from at least 6 hours)

Exclusion criteria:

• patient refusal;

• patients with known bleeding diathesis / ongoing bleeding;

• patients at risk of bleeding (defined as thrombocytes <50 G/l, INR> 2.5, aPTT> 70 sec);

• patients with traumatic brain injury / polytrauma;

• patients with esophagus-tracheal fistulas or malformations of the ENT sphere;

• patients with current or previous radiotherapy of the ENT sphere;

• patients unable to give their informed consent due to language barriers;

• women who are pregnant.

## 5.2 Recruitment, screening and informed consent procedure

In both centers, after the EC approval of the study, all patients fulfilling eligibility criteria (see Section 4.1) will be recruited in daily clinical practice through the Project Leader or principal investigator or their designees.

All patients will receive an information sheet and a study-specific consent form describing the study and providing sufficient information for patient to make an informed decision about his/her participation in the study. The patients should not sign the informed consent on the same day when it is delivered to him/her to ensure adequate time for reflection and discussion with relatives or other trusted persons.

Each patient will be informed that the participation in the study is voluntary and that he/she may withdraw from the study at any time and that withdrawal of consent will not affect her subsequent medical assistance and treatment. The patient must also be informed that his/her medical records may be examined by authorized individuals other than their treating physician.

The patient information sheet and the consent form will be submitted to the EC to be reviewed and approved. The formal consent of a patient, using the approved consent form, must be obtained before the patient undergoes the procedures of the study.

The patient should read and consider the statement before signing and dating the informed consent form, and should be given a copy of the signed document. The consent form must also be signed and dated by the investigator (or his designee) and it will be retained as part of the study records.

##

## 5.3 Withdrawal and discontinuation

Patients will be taken off study if they withdraw their consent to participate. In such case data collected until the date of withdraw will be used

## 5.4 Project Schedule

See Table 1 in appendix.

## 5.5 Study procedures

Peri-procedural evaluations

• Before anaesthesia;

• Patient eligibility (inclusion / exclusion criteria);

• Patient registration and recording of clinical and demographic data (height, weight, BMI);

• Anonymization by alpha / numeric code;

• Vital parameters recording: non-invasive arterial pressure, heart rate, respiratory rate.

- Group A patients (Clinica Luganese Moncucco):

• Clinical parameters recorded: COPD diagnosis, pulmonary embolism heart disease;

• Study parameters recorded: endotracheal etCO2, esophageal etCO2.

- Group B patients (Regional Hospital of Bellinzona):

• Clinical parameters: diagnosis of jatal hernia, intake of proton pump inhibitors (PPI), fasting hours, presence of reflux disease, enteral feeding;

• Study parameters: esophageal pH (25 cm from the dental arch), gastric pH (40 cm from the dental arch).

Study duration: 1 year (there is no follow up), including a recruitment period of 12 months

# STATISTICS AND METHODOLOGY

## 6.1 Determination of sample size

Previous studies allow us to estimate an etCO2 tracheal value of around 40 mmHg (CI 30-50 mmHg). In order to have a significant difference between tracheal and esophageal groups, with a power of 90% and a significance level of 0.01 (one-tailed z test), we calculate a number of 35 patients (57). Anticipating a 10% in the registration failure rate, it appears useful to register at least 40 patients for group A. In the same way we estimate an esophageal pH level value around 7 (CI 5 – 8); in order to have a significant difference between esophageal and gastric pH value, with a power of 90% and a significance level of 0.01 (one-tailed z test), we calculate a number of 30 patients. Anticipating a 10% of drop off rate, it appears useful to register at least 35 patients for each measurement of group B.

## 6.2. Statistical analysis plan

We tabulate the distribution of baseline variables across the study sections, summarizing categorical variables by frequencies and percentage and numerical variables either by mean and standard deviations (±SDs), or by medians and interquartile ranges (IQR). Data distribution was verified using a Kolmogorov- Smirnov test.

We checked adherence to the protocol for each patient during the study. We assess and report total adherence rate to our protocol in order to analyse data on the intention-to-treat (ITT) and on the per-protocol (PP) population. Primary analysis is carried out on the ITT population for each study group, irrespective of potential non-adherence to the study protocol. Secondary analyses included analysis on the PP population, concerning the patients enrolled and treated following all the study procedures. The results of the performed analyses on the two populations are compared. All hypothesis tests are one-sided and considered significant if p-value is ≤ 0.01. Analyses are accomplished with the intention-to-treat if not otherwise specified.

In group A we tested the hypothesis of difference in the proportion of etCO2 level into the tracheal group and into the esophageal group (measurements collected in patients enrolled in group A), with the ITT. We executed a z-test for comparison of the two proportions, refusing the null hypothesis of no difference between the two if the p-value was ≤0.01. This to identify the threshold value of etCO2 above which tracheal location of NGT is determined with high precision. Similarly, in group B we tested the hypothesis of difference in the proportion of pH level into the esophageal and gastric group, with the ITT. We carried out a z-test for comparison of the two proportions, refusing the null hypothesis of no difference between the two if the p-value was ≤ 0.01. This to identify the threshold value of pH below which gastric location of NGT is determined with high precision. Statistical analysis was performed using SPSS version 10.0 (IBM, Chicago, IL, USA) for Mac OS.

# 7 Regulatory Aspects AND SAFETY

## 7.1 Local regulations / Declaration of Helsinki

This research project will be conducted in accordance with the protocol, the Declaration of Helsinki, the principles of Good Clinical Practice, the Human Research Act (HRA) and the Human Research Ordinance (HRO) as well as other locally relevant regulations.

## 7.2 Serious Events

If a serious event occurs, the research project will be interrupted and the Ethics Committee notified on the circumstances via BASEC within 7 days according to HRO Art. 21.

## 7.3 Amendments

Substantial changes to the project set-up, the protocol and relevant project documents will be submitted to the Ethics Committee for approval according to HRO Art. 18 before implementation. Exceptions are measures that have to be taken immediately in order to protect the participants.

## 7.4 End of the project

Upon project termination, the Ethics Committee is notified within 90 days. See Section 9.4 for further details on how health-related data will be handled upon termination of the project.

## 7.5 Insurance

The research project is of category A according to HRO Art. 7. No insurance would be required for this study.

# 8 FURTHER Aspects

## 8.1 Overall ethical considerations

The Project Leader affirms and upholds the principle of the participants’ right to dignity, privacy and health and that the project team shall comply with applicable privacy laws. Especially, anonymity of the participants shall be guaranteed when presenting the data at scientific meetings or publishing them in scientific journals.

Individual participant medical information obtained as a result of this research project is considered confidential and disclosure to third parties is prohibited. Participant confidentiality will be further ensured by utilizing identification code numbers to correspond to medical information in the computer files.

For data verification purposes, a competent authority or an ethics committee may require direct access to parts of the medical records relevant to the project, including participants’ medical history.

## 8.2 Risk-benefit assessment

This study consists in several parameters collection in patients who required a NGT positioning. Thus, any potential clinical risk is related only to the standard procedure of NGT placing.

There will be no immediate benefits to the enrolled patients. Future patients who required a NGT positioning could benefit due to the potential of using threshold values as predictors of correct NGT placing, offering better treatment approach which not required thoracic-abdominal anterior-posterior radiography as diagnostic gold standard.

# 9 Quality CONTROL AND Data protection

## 9.1 Quality measures

The project will be conducted according to the ICH-GCP and national requirements. Quality assurance and control will follow the Project Leader’s SOPs.

The Project Leader is responsible for proper training of all personnel delegated to perform study-related activities and for supervising the conduct of the study.

All collected data will be double-checked by the Project Leader or delegated person, different from the one who entered the data, to ensure accuracy and consistency; correction will be done accordingly.

## 9.2 Data recording and source data

The physicians will maintain individual records of each patient. These records constitute source data. Case Report Form (CRF) specifically created for this study will be used to collect clinical data. The CRFs have to be completed in a timely manner. All source data will be kept according to all applicable regulatory requirements.

Only coded data will be entered in the CRF and these will be stored at the Clinical Research Unit of Clinica Luganese Moncucco. The access is restricted only to authorized staff. A patient identification list must be maintained in order to allow identification of a patient. Coding keys is stored at the project leader site. Only persons involved in the study will have access to the key.

## 9.3 Confidentiality and coding

Study and participant data will be handled with uttermost discretion and is only accessible to authorized personnel who require the data to fulfil their duties within the scope of the study. On all project specific documents, participants are only identified by a unique participant number.

## 9.4 Retention and destruction of study data and biological material

All project data and relevant source documents will be maintained at the Project’s leader site for a minimum of 10 years after study termination or premature termination of the project.

# 10. Funding / Publication / declaration of Interest

We will expect a financial support of around 3500 CHF from Nestlé. The confirmation will be given in the next few weeks (mid-December).

The Project Leader has the responsibility of the collected data.

In case of a project grant, the company will have none property on the collected data.

The Project Leader and all investigators involved into the study have no conflict of interest to declare.

The projects results are planned to be reported in an original article to be submitted to a peer reviewed scientific journal.

# REFERENCES

1. Aronchik J, Epstein D, Gefter W, et al. Pneumothorax as a complication of placement of a nasoenteric tube. JAMA 1984;252:3287-8.
2. Asai T, Stacey M: Confirmation of feeding tube position: How about capnography? Anaesthesia 1994; 49:451
3. Balogh G, Adler S, Vander Woude J, et al. Pneumothorax as a complication of feeding tube placement. Am J Radiol 1983;141:1275-7.
4. Bercik P1, Schlageter V, Mauro M, Rawlinson J, Kucera P, Armstrong D. Noninvasive verification of nasogastric tube placement using a magnet-tracking system: a pilot study in healthy subjects. JPEN J Parenter Enteral Nutr. 2005 Jul-Aug;29(4):305-10.
5. Bohnker BK, Artman LE, Hoskins WJ: Narrow bore nasogastric feeding tube complications. Nutr Clin Pract 2:203–209, 1987.
6. Boyes RJ, Kruse JA: Nasogastric and nasoenteric intubation. Crit Care Clin 8:865–878, 1992.
7. Burns SM, Carpenter R, Truwitt JD: Report of development of a procedure to prevent placement of feeding tubes into the lungs using end-tidal CO2 measurements. Crit Care Med 2001; 29:936 –939
8. Chang J, Melnick B, Bedger R, et al. Inadvertent endobronchial intubation with nasogastric tube. Arch Otolaryngol 1982;108:528-9.
9. Chun DH, Kim NY, Shin YS, Kim SH. A randomized, clinical trial of frozen versus standard nasogastric tube placement. World J Surg. 2009 Sep;33(9):1789-92
10. Common Terminology Criteria for Adverse Events (CTCAE)
    https://www.eortc.be/services/doc/ctc/CTCAE_4.03_2010-06-14_QuickReference_5x7.pdf
11. D’Souza CR, Kilam SA, D’Souza U, Janzen EP, Sipos RA: Pulmonary complications of feeding tubes: a new technique of insertion and monitoring malposition. Can J Surg 1994;37(5):404–408.
12. Declaration of Helsinki
    https://www.wma.net/policies-post/wma-declaration-of-helsinki-ethical-principles-for-medical-research-involving-human-subjects/
13. Dobranowski J, Fitzgerald J, Baxter F, et al: Incorrect positioning of nasogastric feeding tubes and the development of pneumothorax. Can Assoc Radiol J 1992; 43:35–39
14. Dorsey J, Cogordan J. Nasotracheal intubation and pulmonary parenchymal perforation. Chest 1985;87:131-2.
15. Elpern, E.H., Killeen, K., Talla, E., Perez, G., Gurka, D. Capnometry and air insufflation for assessing initial placement of gastric tubes. American Journal of Critical Care 2007;16(6):544–549.
16. Federal Act on Data Protection (FADP)
    https://www.admin.ch/opc/en/classified-compilation/19920153/index.html
17. Fernandez RS, Chau JP, Thompson DR, Griffiths R, Lo HS. Accuracy of biochemical markers for predicting nasogastric tube placement in adults--a systematic review of diagnostic studies. Int J Nurs Stud. 2010 Aug;47(8):1037-46
18. Frakes, M.A.. Measuring end-tidal carbon dioxide: clinical applications and usefulness. Critical Care Nurse 2001;21(5):23–35.
19. Ghahremani GG, Gould RJ: Nasoenteric feeding tubes. Radiographic detection of complications. Dig Dis Sci 31:574–585, 1986.
20. Gilbertson HR, Rogers EJ, Ukoumunne OC. Determination of a practical pH cutoff level for reliable confirmation of nasogastric tube placement. JPEN J Parenter Enteral Nutr. 2011 Jul;35(4):540-4
21. Grossman T, Duncavage J, Kay J, et al. Complications associated with a narrow-bore nasogastric tube. Ann Otol Rhinol Laryngol 1984;93:460-3.
22. Hand R, Kempster M, Levy J, et al. Inadvertent transbronchial insertion of narrow-bore feeding tubes. JAMA 1984;251:2396-7.
23. Harris MR, Huseby JS: Pulmonary complications from nasoenteral feeding tube insertion in an intensive care unit: Incidence and prevention. Crit Care Med 1989; 18:917–919
24. Hendry P, Akyurekli Y, McIntryre, et al. Bronchopleural complications of nasogastric feeding tubes. Crit Care Med 1986;14:892-4.
25. Hess DR: Capnometry. In: Principles and Practice of Intensive Care Monitoring. Tobin MJ (Ed). New York, McGraw-Hill, 1998, pp 377– 400
26. Higgins, J.P., Thompson, S.G., Deeks, J.J., Altman, D.G. Measuring inconsistency in meta-analyses. British Medical Journal 2003;327:557–560.
27. Howes, D.W., Shelley, E.S., Pickett, W. Colorimetric carbon dioxide detector to determine accidental tracheal feeding tube placement. Canadian Journal of Anaesthesia 2005;52(4):428–432.
28. Human Research Act (HRA)
    https://www.admin.ch/opc/de/classified-compilation/20061313/index.html
29. International Conference on Harmonization (ICH) E2A Clinical Safety Data Management: Definitions and Standards for Expedited Reporting
    http://www.ema.europa.eu/docs/en_GB/document_library/Scientific_guideline/2009/09/WC500002749.pdf
30. International Conference on Harmonization (ICH) E6(R2) Guideline for Good Clinical Practice
    http://www.ich.org/fileadmin/Public_Web_Site/ICH_Products/Guidelines/Efficacy/E6/E6_R2__Step_4_2016_1109.pdf
31. Jaffe, M.B. Carbon dioxide measurement. In: Gravenstein, J.S., Jaffe, M.B., Paulus, D.A. (Eds.), Capnography: Clinical Aspects: Carbon Diox- ide over Time and Volume. Cambridge University Press, Cambridge, UK, 2004; pp. 399–412.
32. Kim HM, So BH, Jeong WJ, Choi SM, Park KN. The effectiveness of ultrasonography in verifying the placement of a nasogastric tube in patients with low consciousness at an emergency center. Scand J Trauma Resusc Emerg Med. 2012 Jun 12;20:38
33. Kindopp, A.S., Drover, J.W., Heyland, D.K. Capnography confirms correct feeding tube placement in intensive care unit patients. Canadian Journal of Anaesthesia 2001;48(7):705–710.
34. Kiwak M, McLoud T, Dedrick C, et al. Entriflex feeding tube: need for care in using it. Am J Radiol 1984;143:1341-2.
35. Leder, S.B., Suiter, D.M. Effect of nasogastric tubes on incidence of aspiration. Archives of Physical Medicine and Rehabilitation 2008;89(4):648–651.
36. Lipman TO, Kessler T, Arabian A: Nasopulmonary intubation with feeding tubes: Case reports and review of the literature. JPEN J Parenter Enteral Nutr 1985; 9:618 – 620
37. Liu S.Y., Lee T.S. Bongard F. Accuracy of capnography in nonitubated surgical patients. Chest 1992 102(5), pp. 1512-1515
38. Marderstein EL, Simmons RL, Ochoa JB. Patient safety: effects of institutional protocols on adverse events related to feeding tube placement in the critically ill. J Am Coll Surg. 2004;199(1):39-50.
39. Mercurio P, Levine P: Determining NG tube position. (Letter). Respir Care 1985;30:999.
40. Metheny NA, McSweeney M, Wehrle M, et al: Effectiveness of the auscultatory method in predicting feeding tube location. Nurs Res 1990; 39:262–267
41. Metheny NA, Reed L, Wiersema L, et al: Effectiveness of pH measurements in predicting feeding tube placement: An update. Nurs Res 1993; 42:324 –331
42. Metheny NA, Stewart BJ, Smith L, et al: pH and concentration of bilirubin in feeding tube aspirates as predictors of tube placement. Nurs Res 1999; 48:189 –197
43. Metheny NA, Stewart BJ, Smith L, et al: pH and concentrations of pepsin and trypsin in feeding tube aspirates as predictors of tube placement. J Parenter Enteral Nutr 1997; 21:279 –285
44. Meyer, P., Henry, M., Maury, E., Baudel, J.L., Guidet, B., Offenstadt, G. Colorimetric capnography to ensure correct nasogastric tube position. Journal of Critical Care 2009;24(2):231–235.
45. Nakao MA, Killman D, Wilson R: Pneumothorax secondary to inadvertent nasotracheal placement of a nasoenteric tube past a cuffed endotracheal tube. Crit Care Med 1983; 11: 210 –211
46. Nellcor Puritan Bennett, Inc., 2005. CO_2_ Detectors. Easy Cap II/Pedi-Cap. Nellcor Puritan Bennett, Inc., Pleasanton, CA. (accessed 01.07.09.)http://www.nellcor.com/_Catalog/PDF/Product/EasyCapII- SalesCard.pdf.
47. Nguyen L, Lewiss RE, Drew J, Saut T. A novel approach to confirming nasogastric tube placement in the ED. Am J Emerg Med. 2012 Oct;30(8):1662.e5-7
48. Ordinance on Clinical Trials in Human Research (ClinO)
    https://www.admin.ch/opc/de/classified-compilation/20121176/index.html
49. Ozer S1, Benumof JL. Oro- and nasogastric tube passage in intubated patients: fiberoptic description of where they go at the laryngeal level and how to make them enter the esophagus. Anesthesiology. 1999 Jul;91(1):137-43.
50. Portney, L.G., Watkins, M.P. Foundations of Clinical Research: Applications to Practice, 2nd ed. 2000 Prentice Hall Health, Upper Saddle River.
51. Prasad G, Garg R. The 'bubble technique': an innovative technique for confirming correct nasogastric tube placement. J Clin Anesth. 2011 Feb;23(1):84-5
52. Rakel, B.A., Titler, M., Goode, C., Barry-Walker, J., Budreau, G., Buckwalter, K.C. Nasogastric and nasointestinal feeding tube placement: an integrative review of research. AACN Clinical Issues in Critical Care Nursing 1994;5(2):194–206.
53. Randall HT: History of enteral feeding: Past and present perspectives. In: Clinical Nutrition: Enteral and Tube Feeding. Rombeau JL, Kersey R, Rolandelli R (Eds). St. Louis, MO, Saunders, 1996, pp 1–11
54. Roubenhoff R, Raviech WJ: Pneumothorax due to nasogastric feeding tubes. Report of four cases, review of the literature, and recommendations for prevention. Arch Intern Med 1989; 149:184–188
55. Saltzberg DM, Goldstein M, Levine GM: Feeding tube-induced pneumothorax. JPEN J Parenter Enteral Nutr 1984;8:714–716
56. Sanaka, M., Kishida, S., Yoritaka, A., Sasamura, Y., Yamamoto, T., Kuyama, Y. Acute upper airway obstruction induced by an indwelling long intestinal tube: attention to the nasogastric tube syndrome. Journal of Clinical Gastroenterology 2004;38(10):913.
57. Simel, D.L., Samsa, G.P., Matchar, D.B. Likelihood ratios with con- fidence: sample size estimation for diagnostic test studies. Journal of Clinical Epidemiology 1991;44(8):763–770.
58. St. John RE: End-Tidal CO_2_ Monitoring. Aliso Viejo, CA: American Association of Critical Care Nursing Publications, 1996. Protocols for Practice: Non-Invasive Monitoring Series.
59. Stein N., Matz H., Scheeweib A., Eckmann C., Roth-Isigkeit A., Huppe M., Gehring H. An Evaluation of a Transcutaneous and an End-Tidal Capnometer for Noninvasive Monitoring of Spontaneously Breathing Patients. Respiratory Care 2006 51(10), pp. 1162-1166
60. Swiech, K., Lancaster, D.R., Sheehan, R. Use of a pressure gauge to differentiate gastric from pulmonary placement of nasoenteral feeding tubes. Applied Nursing Research 1994;7(4):183–189.
61. Thomas, B.W., Falcone, R.E. Confirmation of nasogastric tube placement by colorimetric indicator detection of carbon dioxide: a preliminary report. Journal of the American College of Nutrition 1998;17(2):195–197.
62. Tobin, R.W., Gonzales, A.J., Golden, R.N., Brown, M.C., Silverstein, F.E., 2000. Magnetic detection to position human nasogastric tubes. Biomedical Instrumentation and Technology 2000;34(6):432–436.
63. Tornero C, Herrejon A, Salcedo M. Pneumothorax, atelectasis, and pleural effusion secondary to the placement of an enteral feeding tube. Rev Clin Esp 1992;191:286–287
64. Torrington KG, Bowman MA: Fatal hydrothorax and empyema complicating a malpositioned nasogastric tube. Chest 1981; 79: 240 –242
65. Valentine RJ, Turner WW Jr: Pleural complications of nasoenteric feeding tubes. J Parenter Enteral Nutr 1985;9:605–607.
66. Vigneau C, Baudel JL, Guidet B, Offenstadt G, Maury E. Sonography as an alternative to radiography for nasogastric feeding tube location. Intensive Care Med. 2005 Nov;31(11):1570-2
67. Wendell GD, Lecchner GS, Promisloff RA: Pneumothorax complicating small bore feeding tube placement. Arch Intern Med 1991; 151:599 – 602
68. Woodall BH, Winfield DF, Bisset GS III: Inadvertent tracheobronchial placement of feeding tubes. Radiology 1987;165:727–729
69. Wu, P.Y., Kang, T.J., Hui, C.K., Hung, M.H., Sun, W.Z., Chan, W.H. Fatal massive hemorrhage caused by nasogastric tube misplacement in a patient with mediastinitis. Journal of the Formosan Medical Association 2006;105 (1):80–85.

# Appendix 1: Tab. 1

| **Time (day,)** | **>-7 days** | **0** | **+1** |
| --- | --- | --- | --- |
| **Visit** | Information | Screening | Pose of NGT |
| **Oral and written patient information** | + |  |  |
| **Written consent** |  | + |  |
| **Inclusion-/**  **exclusion criteria** |  | + |  |
| **Medical history** |  | + |  |
| **Participant characteristics** |  | + |  |
| **Procedures** |  |  | + |
| **Sampling** |  |  | + |
| **Safety** |  | + | + |
