# Supplementary material for: Nasogastric tube in mechanical ventilated patients: ETCO2 and pH measuring to confirm correct placement. A pilot study
Source: PLoS One. 2022 Jun 2;17(6):e0269024. doi: 10.1371/journal.pone.0269024 (PMC9162373; doi:10.1371/journal.pone.0269024)
Supplement: Attachment — Submitted filename: PONE-D-21-20567-FB PR.pdf [file pone.0269024.s005.pdf]

PLOS ONE

# Nasogastric tube in critical care setting: combining ETCO2 and pH measuring to confirm correct placement

--Manuscript Draft--

**Manuscript Number:** PONE-D-21-20567

**Article Type:** Clinical Trial

**Full Title:** Nasogastric tube in critical care setting: combining ETCO2 and pH measuring to confirm correct placement

**Short Title:** Nasogastric tube in critical care setting

**Corresponding Author:** Samuele Ceruti
Clinica Luganese Moncucco
Lugano, Ticino SWITZERLAND

**Keywords:** gastric pH, intubation, capnography, capnography / methods, Intensive Care Units

**Abstract:**

exposure

Introduction

Nasogastric tube (NGT) placement is a common procedure performed in critical care setting. Chest X-Ray is the diagnostic gold-standard to confirm correct placement, with the downsides of both the need for critical care patients' mobilization and intrinsic actinic risk. Other potential methods to confirm NGT placement have shown lower accuracy compared to chest X-ray; ETCO2 and pH analysis have singularly yet investigated as an alternative to the gold standard. Aim of this study was to determine thresholds in combine measurements of ETCO2 and pH values, at which correct NGT positioning can be confirmed with the highest accuracy.

Material & Methods

A prospective, multicenter, observational trial; a continuous cohort of eligible patients was allocated to two arms, to identify clear cut-off threshold able to detect correct NGT tip positioning with the maximal accuracy. Patients underwent general anesthesia and orotracheal intubation; in the first group difference between tracheal and esophageal ETCO2 values were assessed. In the second group difference between esophageal and gastric pH values were determined.

Results

From November 2020 to March 2021, 85 consecutive patients were enrolled: 40 in the ETCO2 group and 45 in the pH group. The ETCO2 ROC analysis for predicting NGT tracheal misplacement demonstrate an optimal ETCO2 cutoff value of 25.5 mmHg, where both sensitivity than specificity reach 1.0 (AUC 1.0, $p < 0.001$). The pH ROC analysis for predicting NGT correct gastric placement demonstrated the optimal pH cutoff value at 4.25, with a mild diagnostic accuracy (AUC 0.79, $p < 0.001$).

Discussion

A device capable of combining the presence of a negative marker with a positive marker could be accurate enough in identifying the correct NGTs positioning. Further studies are required to validate the reproducibility of these results by a specific device, whose accuracy also ought to be compared with standard chest X-ray.

**Order of Authors:**
Samuele Ceruti
Simone Dell'Era
Francesco Ruggiero
Giovanni Bona
Andrea Glotta
Maira Biggiogero

*Powered by Editorial Manager® and ProduXion Manager® from Aries Systems Corporation*

| | |
|---|---|
| | Edoardo Tasciotti |
| | Christoph Kronenberg |
| | Andrea Saporito |
| **Additional Information:** | |
| **Question** | **Response** |
| **Financial Disclosure**<br><br>Enter a financial disclosure statement that describes the sources of funding for the work included in this submission. Review the submission guidelines for detailed requirements. View published research articles from *PLOS ONE* for specific examples.<br><br>This statement is required for submission and **will appear in the published article** if the submission is accepted. Please make sure it is accurate.<br><br>**Unfunded studies**<br>Enter: *The author(s) received no specific funding for this work.*<br><br>**Funded studies**<br>Enter a statement with the following details:<br>• Initials of the authors who received each award<br>• Grant numbers awarded to each author<br>• The full name of each funder<br>• URL of each funder website<br>• Did the sponsors or funders play any role in the study design, data collection and analysis, decision to publish, or preparation of the manuscript?<br>• **NO** - Include this sentence at the end of your statement: *The funders had no role in study design, data collection and analysis, decision to publish, or preparation of the manuscript.*<br>• **YES** - Specify the role(s) played.<br><br>* typeset | The authors received no specific funding for this work. |
| **Competing Interests**<br><br>Use the instructions below to enter a competing interest statement for this submission. On behalf of all authors, disclose any competing interests that could be perceived to bias this | The authors have declared that no competing interests exist |

| | |
|---|---|
| work—acknowledging all financial support and any other relevant financial or non-financial competing interests.<br><br>This statement is required for submission and **will appear in the published article** if the submission is accepted. Please make sure it is accurate and that any funding sources listed in your Funding Information later in the submission form are also declared in your Financial Disclosure statement.<br><br>View published research articles from *PLOS ONE* for specific examples.<br><br>**NO authors have competing interests**<br><br>Enter: *The authors have declared that no competing interests exist.*<br><br>**Authors with competing interests**<br><br>Enter competing interest details beginning with this statement:<br><br>*I have read the journal's policy and the authors of this manuscript have the following competing interests: [insert competing interests here]*<br><br>* typeset | |
| **Ethics Statement**<br><br>Enter an ethics statement for this submission. This statement is required if the study involved:<br><br>• Human participants<br>• Human specimens or tissue<br>• Vertebrate animals or cephalopods<br>• Vertebrate embryos or tissues<br>• Field research<br><br>Write "N/A" if the submission does not require an ethics statement. | Comitato Etico Cantonale, Bellinzona, Switzerland - N. CE3548<br>Written informed consent form |

*Powered by Editorial Manager® and ProduXion Manager® from Aries Systems Corporation*

| | |
|---|---|
| General guidance is provided below. Consult the submission guidelines for detailed instructions. **Make sure that all information entered here is included in the Methods section of the manuscript.**<br><br>**Format for specific study types**<br><br>**Human Subject Research (involving human participants and/or tissue)**<br>• Give the name of the institutional review board or ethics committee that approved the study<br>• Include the approval number and/or a statement indicating approval of this research<br>• Indicate the form of consent obtained (written/oral) or the reason that consent was not obtained (e.g. the data were analyzed anonymously)<br><br>**Animal Research (involving vertebrate animals, embryos or tissues)**<br>• Provide the name of the Institutional Animal Care and Use Committee (IACUC) or other relevant ethics board that reviewed the study protocol, and indicate whether they approved this research or granted a formal waiver of ethical approval<br>• Include an approval number if one was obtained<br>• If the study involved *non-human primates*, add *additional details* about animal welfare and steps taken to ameliorate suffering<br>• If anesthesia, euthanasia, or any kind of animal sacrifice is part of the study, include briefly which substances and/or methods were applied<br><br>**Field Research**<br><br>Include the following details if this study involves the collection of plant, animal, or other materials from a natural setting:<br>• Field permit number<br>• Name of the institution or relevant body that granted permission | approval not stated here<br><br>why, please explain |
| **Data Availability**<br><br>Authors are required to make all data underlying the findings described fully | No - some restrictions will apply |

*Powered by Editorial Manager® and ProduXion Manager® from Aries Systems Corporation*

| | |
|---|---|
| available, without restriction, and from the time of publication. PLOS allows rare exceptions to address legal and ethical concerns. See the PLOS Data Policy and FAQ for detailed information.<br><br>A Data Availability Statement describing where the data can be found is required at submission. Your answers to this question constitute the Data Availability Statement and **will be published in the article**, if accepted.<br><br>**Important:** Stating 'data available on request from the author' is not sufficient. If your data are only available upon request, select 'No' for the first question and explain your exceptional situation in the text box.<br><br>Do the authors confirm that all data underlying the findings described in their manuscript are fully available without restriction? | |
| **Describe where the data may be found in full sentences. If you are copying our sample text, replace any instances of XXX with the appropriate details.**<br><br>• If the data are **held or will be held in a public repository**, include URLs, accession numbers or DOIs. If this information will only be available after acceptance, indicate this by ticking the box below. For example: *All XXX files are available from the XXX database (accession number(s) XXX, XXX.*).<br>• If the data are all contained **within the manuscript and/or Supporting Information files**, enter the following: *All relevant data are within the manuscript and its Supporting Information files.*<br>• If neither of these applies but you are able to provide **details of access elsewhere**, with or without limitations, please do so. For example:<br><br>*Data cannot be shared publicly because of [XXX]. Data are available from the XXX Institutional Data Access / Ethics* | The datasets used and/or analyzed during the current study available from the corresponding author on reasonable request.<br><br>"reasonable request" is too vague; prefer the use of onthe editor's samplese |

| | |
|---|---|
| *Committee (contact via XXX) for researchers who meet the criteria for access to confidential data.*<br><br>*The data underlying the results presented in the study are available from (include the name of the third party and contact information or URL).*<br>• This text is appropriate if the data are owned by a third party and authors do not have permission to share the data.<br><br>* typeset | |
| Additional data availability information: | Tick here if the URLs/accession numbers/DOIs will be available only after acceptance of the manuscript for publication so that we can ensure their inclusion before publication. |

Powered by Editorial Manager® and ProduXion Manager® from Aries Systems Corporation

Manuscript

Click here to access/download;Manuscript;Ceruti_PLOS_II.docx

# Nasogastric tube in critical care setting: combining ETCO2 and pH measuring to confirm correct placement

## Short title: Nasogastric tube in critical care setting

S. Ceruti[1]*, S. Dell'Era [2], F. Ruggiero[3], G. Bona[4], A. Glotta[1], M. Biggiogero[4], E. Tasciotti[2], C. Kronenberg[2], A. Saporito[2]

[1] Department of Critical Care, Clinica Luganese Moncucco, Lugano, Ticino, Switzerland

[2] Service of Anesthesiology, Ospedale Regionale di Bellinzona e Valli, Bellinzona, Ticino, Switzerland

[3] Department of Internal Medicine, Clinica Luganese Moncucco, Lugano, Ticino, Switzerland

[4] Clinical Research Unit, Clinica Luganese Moncucco, Lugano, Ticino, Switzerland

*** CORRESPONDING AUTHOR:**

E-mail: samuele.ceruti@moncucco.ch (SC)

# Abstract

**Introduction:** nasogastric tube (NGT) placement is a common procedure performed in critical care setting. Chest X-Ray is the diagnostic gold-standard to confirm correct placement, with the downsides of both the need for critical care patients' mobilization and intrinsic actinic risk. Other potential methods to confirm NGT placement have shown lower accuracy compared to chest X-ray; $ETCO_2$ and pH analysis have singularly yet investigated as an alternative to the gold standard. Aim of this study was to determine thresholds in combine measurements of $ETCO_2$ and pH values, at which correct NGT positioning can be confirmed with the highest accuracy.

**Material & Methods:** a prospective, multicenter, observational trial; a continuous cohort of eligible patients was allocated to two arms, to identify clear cut-off threshold able to detect correct NGT tip positioning with the maximal accuracy. Patients underwent general anesthesia and orotracheal intubation; in the first group difference between tracheal and esophageal $ETCO_2$ values were assessed. In the second group difference between esophageal and gastric pH values were determined.

**Results:** from November 2020 to March 2021, 85 consecutive patients were enrolled: 40 in the $ETCO_2$ group and 45 in the pH group. The $ETCO_2$ ROC analysis for predicting NGT tracheal misplacement demonstrate an optimal $ETCO_2$ cutoff value of 25.5 mmHg, where both sensitivity than specificity reach 1.0 (AUC 1.0, $p < 0.001$). The pH ROC analysis for predicting NGT correct gastric placement demonstrated the optimal pH cutoff value at 4.25, with a mild diagnostic accuracy (AUC 0.79, $p < 0.001$).

**Discussion:** A device capable of combining the presence of a *negative marker* with a *positive marker* could be accurate enough in identifying the correct NGTs positioning. Further studies are required to validate the reproducibility of these results by a specific device, whose accuracy also ought to be compared with standard chest X-ray.

Trial number: NCT03934515 (www.clinicaltrials.gov)

# Introduction

Nasogastric tube (NGT) placement is an extremely common procedure routinely performed on patients in critical care setting[1]. This simple procedure however is not riskless: serious complications can occur, especially in sedated, intubated and paralyzed patients, when cough reflex have been abolished[2]. The incidence of complications during NGT positioning is around 4%, with both a high morbidity[3], possibly leading to a prolonged hospital stay and higher costs and an increased mortality[4].

To be rephrased

Currently, the gold standard for correct positioning confirmation is the Chest X-Ray[5], which implies the use of ionizing radiation (4 μSv for radiography). This may not be negligible considering that critical patients, during their hospital stay, may require multiple NGT placements and repositioning after displacement. Furthermore, in critically ill patients the image receptor is placed directly under the back, requiring ~~their passive~~ mobilization, which increases both staff workload and clinical risks (for example of accidental extubation or hemodynamic instability).

Several alternatives have been investigated in order to confirm correct NGT placement; these include the so-called *bubble technique*[6], frozen NGT[7], gastric auscultation[8,9], aspiration from the NGT[10,11], gastric ultrasound[12–14], biochemical markers[15,16] and the use of magnets[17]; however none of them was associated to a high diagnostic accuracy. Some pilot studies have shown that measurements of end tidal $CO_2$ ($ETCO_2$) with a graphic capnometer could be used to determine whether NGT tip has been erroneously placed at tracheal level[18–22], others that pH measurements can distinguish a correct NGT tip position from an esophageal positioning, by analyzing the relative pH levels[23,24]. In those studies, however, threshold values of $ETCO_2$ and pH able to discriminate between correct and failed positioning have not been determined: the two measurements had never been associated, the global accuracy of each methodology was rather low, or the sample size was

Insert determined: FURTHERMORE the 2 measuremnets have never been COMBINED) ….

insufficient to obtain a statistical significance. The feasibility to use these two parameters in a hypothetical device exploiting a double feedback mechanism to detect correct NGT placement with

justifies, warrants

a high accuracy is an attractive possibility which demands further investigations.

of

Aim of the study is to analyze distributions between tracheal and esophageal $ETCO_2$ values and between gastric and esophageal pH values, in order to identify thresholds values at which the correct positioning of NGT can be confirmed with high accuracy.

# Material & methods

This is a prospective, multicenter, observational trial, conducted in a period of six months in two different acute Hospitals. The study has been registered (clinicaltrials.gov, NCT03934515) and approved by the regional Ethical Committee (Comitato Etico Cantonale, Bellinzona, Switzerland, Chairman Prof. Zanini – N. CE3548). A consecutive cohort of patients undergoing general anesthesia, ~~with orotracheal intubation were enrolled~~; inclusion criteria were patients of both sexes, aged more than 18 years, fasting for at least six hours, who underwent general anesthesia and orotracheal intubation, for whom an oro/naso-tracheal tube ~~positioning~~ was planned according to clinical criteria. Exclusion criteria were patient's refusal or inability to give informed consent, pregnancy, known ongoing gastric or esophageal bleeding, coagulation impairment (defined as thrombocytes < 50 G/L, fibrinogen < 1.0 g/L, INR > 2.5, aPTT > 70 sec tested at the preoperative assessment), history of traumatic brain injury or polytrauma, esophago-tracheal fistulas, esophageal varices, ENT malformations and/or tumors, history of radiotherapy for ENT tumors. Patients in whom pH and/or $ETCO_2$ values were not measurable for technical reasons were excluded and considered as drop-outs.

## Randomization and allocation

Eligible patients were allocated 1:1 to one of the two groups. Group A underwent $ETCO_2$ measurement; group B patients underwent pH measurement. Due to ~~the health emergency defined by the COVID-19 pandemic~~, the Clinica Luganese Moncucco was not able to enroll the total number of patients expected for $ETCO_2$ measurements, as required by the protocol. Only 5 patients were enrolled by the Clinica Luganese Moncucco, ~~they~~ underwent the $ETCO_2$ measurement, while the remaining number of patients expected in this group were enrolled at the Anesthesia Service of Bellinzona and Valli Regional Hospital. As required by the protocol, all patients of group B were enrolled at the Anesthesia Service of Bellinzona and Valli Regional Hospital.

## Group A

After anesthesia induction and orotracheal intubation, a suction probe was inserted within the endotracheal tube and tracheal $ETCO_2$ were measured at 15 seconds through a capnometer connected to the probe. Both the probe and the NGT had the same diameter (12 Fr). After the measurement, secretions were aspirated as usual. Then, NGT was positioned using the standard approach and esophageal $ETCO_2$ was measured at 15 seconds through a capnometer attached to the NGT. After the measurement, the capnometer was disconnected, while the NGT was left in palace as usual. $ETCO_2$ values were registered in the data sheet and subsequently transferred into an anonymized electronic database; two sets of values were therefore obtained for each patient: tracheal and esophageal $ETCO_2$.

## Group B

After anesthesia induction and orotracheal intubation, NGT insertion was performed according to local protocols. pH was than measured using litmus paper on liquid aspirated from the NGT. NGT was progressively inserted, measurements were taken on secretions aspirated from NGT at two different steps at two insertion depths: 25 cm from teeth (esophageal level) and at 40 cm from teeth

(gastric level). If it was not possible to aspirate some fluid, 10 ml of NaCl 0.9% were inserted and then aspired back and pH measurements were taken on this washing fluid. All values were registered and archived as described above; two sets of values were therefore obtained for each patient: esophageal and gastric pH.

## Statistical analysis

According to literature, a tracheal $ETCO_2$ value around 40 mmHg[25–27] was assumed as normal, while a normal esophageal $ETCO_2$ was around 20 mmHg[28]. In order to have a significant difference between tracheal and esophageal groups, with a power of 90% and a significance level of 0.01 (one-tailed z test), we calculated a numerosity of 35 patients. Anticipating a 10% drop-out rate, we included 40 patients for group A. With regard to the pH arm, we assumed an esophageal pH level normal value around 7[29] and gastric pH level ranging from 1.0 to 2.5[30]. In order to have a significant difference between esophageal and gastric pH values, with a power of 90% and a significance level of 0.01 (one-tailed z test), we calculated a numerosity of 30 patients. Anticipating a 10% of drop-out rate, we included 35 patients for each measurement in group B. We tabulated the distribution of baseline variables across the study's sections, summarizing categorical variables by frequencies and percentage and numerical variables either by mean and standard deviations (±SDs), or by medians and interquartile ranges (IQR). Data distribution was verified using a Kolmogorov- Smirnov test. We executed a z-test for comparison of the two proportions, refusing the null hypothesis of no difference between the two if the p-value was $\leq 0.01$. In order to identify the threshold value of $ETCO_2$ which signaled endotracheal positioning of the NGT and the pH value threshold who identified a correct gastric location of NGT with high accuracy, the area under the receiver operating characteristic (ROC) curve was calculated for both $ETCO_2$ and for pH level, calculating the sensitivity, specificity and the likelihood ratios for the optimal cut-off point (CP) of the scale (Youden index and Number Necessary to Diagnose, *J* and *NND* respectively)[31]. Starting from the ROC curve, a "cumulative distribution analysis" (CDA) was performed[32], to better identify the gray zone starting from the

zone defined by the values associated with a sensitivity and a specificity both of 90%[33]. All hypothesis tests were one-tailed and considered significant if p-value was ≤0.01. Statistical analysis was performed using SPSS.26 (IBM, Chicago, IL, USA) for MacOS.

# Results

From November 2020 to March 2021, 85 consecutive patients were enrolled: 40 in the $ETCO_2$ group and 45 in the pH group. 19 dropouts occurred, due to incomplete information sampling during the procedure (such as the impossibility to measure pH); 68 patients were therefore included in the analysis, 33 in the $ETCO_2$ group and 35 in the pH group (Fig. 1). Mean age was 54 years old (min/max 46 – 62) and 36 (55%) were men. All demographic data are reported in Table 1.

**Fig. 1 Patients distribution** Study patients' allocation and distribution.

**Table 1. Demographics characteristic population**

| | **$ETCO_2$ group** | **pH group** | **p value** |
|---|---|---|---|
| **Age** | 54 (13.7) | 60 (9.8) | 0.09 |
| **Sex male** | 22 (66%) | 14 (43%) | |
| **BMI [Kg/m$^2$]** | 25.4 (6) | 30.6 (7.4) | 0.36 |
| **Systolic arterial pressure [mmHg]** | 145 (29) | 152 (28) | 0.36 |
| **Heart rate [bpm]** | 80 (21) | 77 (17) | 0.43 |
| **Respiratory rate [min]** | 14 (3) | 14 (2) | 0.7 |
| **Jatal hernia** | - | 6 (18%) | - |
| **PPI intake** | - | 12 (36%) | - |
| **Esophageal Reflux disease** | - | 13 (39%) | - |
| **COPD** | 7 (21%) | - | - |
| **Pulmonary Embolism** | 1 (3) | - | - |
| **Heart disease** | 5 (15) | - | - |
| **Tracheal value** | 40 (7.1) | - | - |
| **Esophageal value** | 11 (9.3) | 5.1 (1.3) | - |

| | | | |
|---|---|---|---|
| **Gastric value** | - | 3.2 (1.7) | - |

Demographic characteristics and main study measurements. Data distribution were expressed as mean ± SD (min-max) or median (25th – 75th) if they are not normally distributed according to Kolmogorov-Smirnov test.

## Distribution analysis

With regard to the $ETCO_2$ *distribution analysis*, 22 (66%) patients were men, 7 (21%) presented a diagnosis of COPD (4 patients of second degree, 1 patient of third degree); one (3%) patient presented a previous diagnosis of pulmonary embolism. Five (15%) patients had a history of heart disease (two patients with severity NYHA 1, three patients with NYHA 2) (Table 1), all with an EF greater than 50%. Mean tracheal $ETCO_2$ was 40 mmHg (SD 7.14), while mean esophageal $ETCO_2$ resulted 11 mmHg (SD 9.3); a t-test score (Fig. 2) confirmed a significant difference (CI 99%, 24- 33, $p < 0.001$).

**Fig. 2 Tracheal and esophageal ETCO2 distribution** Boxplots: the black bar indicates median $ETCO_2$ (38 mmHg and 14 mmHg respectively), while the blue areas include the interquartile ranges for each group.

Regarding *pH distribution analysis*, 14 (40%) patients were male, 6 (18%) presented a history of hiatal hernia, and 13 (39%) presented a diagnosis of gastroesophageal reflux disease, with 12 (36%) patients receiving PPI therapy at the time of data sampling (Table 1); no patient was on enteral feeding during the analysis. Median gastric pH was 3.1 (1.6 – 4.95), while median esophageal pH resulted 5.15 (4.52 – 6.0); a t-test score confirmed a significant difference (CI 99%, 0.9- 2.9, $p = 0.004$, Fig. 3).

**Fig. 3 Measured of esophageal and gastric pH.** Boxplot distribution in all patients and in patients without PPI use. Regarding the whole group analysis, a t-test score confirmed a significant difference between esophageal and gastric values (CI 99%, 0.9- 2.9, $p = 0.004$). The subgroup analysis involving

patients without PPI showed a greater difference (CI 99%, 1.2 – 3.1, $p < 0.001$) compared the whole group. The black bar indicates median pH, while the blue areas include the interquartile ranges for each group.

A subgroup analysis involving 20 (62.5%) patients without PPI, showed a median gastric pH of 2.45 (1.05 – 4.05) and a median esophageal pH of 5.05 (4.52 – 6.0), with a greater difference of t-test score (CI 99%, 1.2 – 3.1, $p < 0.001$) compared to all patients (Fig. 3). A comparison between the mean esophageal pH value in all patients with the mean esophageal value in patients without PPI resulted not significantly different (5.1 vs 4.9, $p = 0.265$).

## ROC curve analysis

The $ETCO_2$ ROC curve analysis for predicting NGT tracheal misplacement (Fig. 4A) demonstrate a perfect diagnostic accuracy with an AUC of 1.0 (CI 95%, 1.0 to 1.0, $p < 0.001$); the optimal cutoff value resulted in an $ETCO_2$ value greater than 25.5 mmHg (Youden index $J = 1$), where both sensitivity and than specificity reach 1.0. The pH ROC curve analysis for predicting ~~NGT~~ correct gastric placement (Fig. 4B) demonstrated a mild diagnostic accuracy with an AUC of 0.79 (CI 95%, 0.67 to 0.90, $p < 0.001$); the optimal cutoff value was a pH below 4.25 (Youden index $J = 0.593$), with a sensitivity of 0.908 and a specificity of 0.687.

**Fig. 4 ROC curves of $ETCO_2$ and pH method**. Receiver operating characteristic (ROC) curves showing the ability of the $ETCO_2$ method (figure 4A) and pH method (Figure 4B) to respectively identify a tracheal misplacement (ROC AUC 1.0, $p < 0.001$) or a gastric correct placement (ROC AUC 0.79, CI 95% 0.67 – 0.90, $p < 0.001$).

The subgroup analysis involving only patient without PPI confirmed a mild diagnostic accuracy, with an AUC of 0.78 (CI 95%, 0.63 – 0.93, $p = 0.002$) with an optimal cutoff pH value below 3.9 (Youden

index J = 0.6). The NND obtained for the $ETCO_2$ method resulted 1, while the NND for the pH method was 1.68 (1.66 in patients without PPI).

Gray zone plots were drawn throughout CDA curves starting from Youden index (Fig. 5) between the 90% of sensibility and 90% of specificity on the two sigma curves for each method ($ETCO_2$ and pH); for the pH the gray zone lay between 4.25 and 5.7 (Fig. 5) while for the $ETCO_2$ no gray zone was identified, as the tracheal and the esophageal distribution did not cross each other (Fig. 6).

**Fig. 5 *Cumulative Distribution Analysis* of pH detection.** Performed to determine the correct NGT placement with (4A) and without (4B) PPI use. The red line indicates the cutoff limit according to Youden Index (pH below 4.25 and pH below 3.9, with J = 0.593 and J = 0.6 respectively); the gray zone is shown, according with a sensibility than a specificity of 90%.

**Fig. 6 *Cumulative Distribution Analysis* of $ETCO_2$ detection.** Performed to exclude the NGT misplacement. The red line indicates the cutoff limit according to Youden Index (J = 1).

# Discussion

Nasogastric tube placement in sedated and intubated patients is a procedure potentially associated with dangerous complications. The gold standard to assess correct positioning is Chest X-Ray, which exposes patients to mobilization-related complications, such as devices displacement and hemodynamic and respiratory instability, as well as to actinic risk, due to the need of multiple X-rays.

Alternatives, such as pH and $ETCO_2$ measurements, when singularly analyzed, failed to show a superiority compared to chest X-Ray in determining correct NGT tip position[18,23,34], in particular due to lack of identification of an actual threshold value. Our study combines these two techniques, in order to investigate if, when applied together, they can accurately detect a correct positioning of the NGT tip. The use of a double feedback mechanism could in fact prove more accurate than one single technique. In this study, $ETCO_2$ distribution between the trachea and esophagus was evaluated

intended as a potential *negative marker* to detect NGT misplacement in the upper airways; at the same time, pH distribution between stomach and esophagus was evaluated as a potential *positive marker* for NGT correct placement. Significant differences between tracheal and esophageal $ETCO_2$ measurements allowed a complete differentiation in the curve plotting distribution. Based on these results, the use of a qualitative capnometer connected to the NGT and set to detect the threshold value of 25.5 mmHg would be a potentially accurate *negative-marker* mechanism for tracheal NGT placement, with a very high sensitivity, thus avoiding any NGT misplacement.

Concerning the differences in results between gastric and esophageal pH, the distributional differences between the two obtained curves is not neat, especially in case of proton pump inhibitors usage, although extremely low pH values were shown to have a high specificity for gastric NGT placement. Fernandez et al published a review of diagnostic studies to test pH of aspirate fluids using a litmus paper; with this method, they evaluated if the NGT had been correctly positioned. It is to be noted that litmus paper color variation could report a value lower than the actual gastric pH, due to limited the litmus paper insufficient sensitivity[16]. A recent clinical trial by Gilbertson et al identified the cut off pH < 5.5 to distinguish between the correct positioning in the stomach[23]. In comparison with these two studies, in our trial, the threshold pH value able to minimize false positive rate, thus increasing the specificity of this *positive marker,* resulted 4.25. Noticeably, even if specificity for very low pH appears to be high, the oppositely low consequent sensitivity would affect the global test accuracy thus invalidating the *positive marker* mechanism for detection of gastric NGT placement (NND = 1.68), leading to potential misses of correct placement.

Furthermore, analyzing the data based on PPI therapy allows determining an even lower pH threshold for patients not receiving this class of medications (pH of 3.9), however guaranteeing the same accuracy. In practice, a pH threshold of 4.25 would therefore assure an even better specificity in this subgroup of patients.

Based on our study, a device capable of combining the presence of a *negative marker* (such as $ETCO_2$) with a *positive marker* (such as pH) could be accurate enough in identifying the correct positioning of NGTs. Further studies are required in order to validate the reproducibility of these results by a specific device, whose accuracy also ought to be compared with chest X-ray, the current diagnostic gold standard.

This study presented some limitations. This was a preliminary study assessing determined physiological variables; it is still unknown whether a device simultaneously sensing $ETCO_2$ and pH could determine correct NGT placement with high accuracy. The presumed esophageal and gastric NGTs placement have been determined based on the distance of the NGT tip from the teeth; there is not, therefore, complete certainty about NGT tip location. However, NGT placed in esophageal site a 40 cm was considered to be placed into the stomach. Moreover, the accuracy of the pH threshold value for the discrimination between esophageal and gastric NGT positioning resulted suboptimal. The use of a normal saline injection in order to measure pH on the aspirated fluid in case secretions could not be aspirated may have affected pH values in these cases.

# Conclusions

In a critical care setting, the use of a device capable of combine the presence of a negative marker to exclude NGT misplacement (like $ETCO_2$) and a positive marker to confirm correct NGT placement (as pH evaluation) could be quite accurate to improve correct NGT placement in unconscious patients. Further studies in this direction are needed in order to test this hypothesis.

# REFERENCES


1. Seron-Arbeloa. Enteral Nutrition in Critical Care. J Clin Med Res. 2013. doi:10.4021/jocmr1210w

2. Sanaie S, Mahmoodpoor A, Najafi M. Nasogastric tube insertion in anaesthetized patients: A comprehensive review. Anaesthesiology Intensive Therapy. Via Medica; 2017. pp. 57–65. doi:10.5603/AIT.a2017.0001

3. Harris MR, Huseby JS. Pulmonary complications from nasoenteral feeding tube insertion in an intensive care unit: Incidence and prevention. Crit Care Med. 1989;17: 917–919. doi:10.1097/00003246-198909000-00016

4. Information S. Patient Safety Alert NPSA / 2011 / PSA002 : Reducing the harm caused by misplaced nasogastric feeding tubes in adults , children and infants. Event (London). 2011.

5. Lortie MA, Charbonney E. Confirming placement of nasogastric feeding tubes. Cmaj. 2016;188: e96. doi:10.1503/cmaj.150609

6. Prasad G, Garg R. The “bubble technique”: An innovative technique for confirming correct nasogastric tube placement. Journal of Clinical Anesthesia. 2011. pp. 84–85. doi:10.1016/j.jclinane.2010.03.006

7. Chun DH, Kim NY, Shin YS, Kim SH. A randomized, clinical trial of frozen versus standard nasogastric tube placement. World J Surg. 2009;33: 1789–1792. doi:10.1007/s00268-009-0144-x

8. Metiieny N. Re: ‘effectiveness of the auscultatory method in predicting feeding tube location’ response. Nurs Res. 1992;41: 189. doi:10.1097/00006199-199205000-00013

9. Simons SR, Abdallah LM. Bedside assessment of enteral tube placement: Aligning practice

with evidence. American Journal of Nursing. 2012. pp. 40–46. doi:10.1097/01.NAJ.0000411178.07179.68

10. Zimmaro Bliss D. pH and Concentration of Bilirubin in Feeding Tube Aspirates As Predictors of Tube Placement NA Metheny, BJ Stewart, L Smith et al. Nursing Research 48(4):189-196, 1999. J Parenter Enter Nutr. 2000;24: 187–188. doi:10.1177/0148607100024003187

11. Metheny NA, Stewart BJ, Smith L, Yan H, Diebold M, Clouse RE. pH and concentrations of pepsin and trypsin in feeding tube aspirates as predictors of tube placement. J Parenter Enter Nutr. 1997;21: 279–285. doi:10.1177/0148607197021005279

12. Nguyen L, Lewiss RE, Drew J, Saul T. A novel approach to confirming nasogastric tube placement in the ED. Am J Emerg Med. 2012;30: 1662.e5-1662.e7. doi:10.1016/j.ajem.2011.09.010

13. Kim HM, So BH, Jeong WJ, Choi SM, Park KN. The effectiveness of ultrasonography in verifying the placement of a nasogastric tube in patients with low consciousness at an emergency center. Scand J Trauma Resusc Emerg Med. 2012;20: 1. doi:10.1186/1757-7241-20-38

14. Vigneau C, Baudel JL, Guidet B, Offenstadt G, Maury E. Sonography as an alternative to radiography for nasogastric feeding tube location. Intensive Care Med. 2005;31: 1570–1572. doi:10.1007/s00134-005-2791-1

15. Turgay AS, Khorshid L. Effectiveness of the auscultatory and pH methods in predicting feeding tube placement. J Clin Nurs. 2010;19: 1553–1559. doi:10.1111/j.1365-2702.2010.03191.x

16. Fernandez RS, Chau JPC, Thompson DR, Griffiths R, Lo HS. Accuracy of biochemical markers for predicting nasogastric tube placement in adults-A systematic review of diagnostic

studies. Int J Nurs Stud. 2010;47: 1037–1046. doi:10.1016/j.ijnurstu.2010.03.015

17. Tobin RW, Gonzales AJ, Golden RN, Brown MC, Silverstein FE. Magnetic detection to position human nasogastric tubes. Biomed Instrum Technol. 2000;34: 432–436.

18. Burns SM, Carpenter R, Truwit JD. Report on the development of a procedure to prevent placement of feeding tubes into the lungs using end-tidal CO2 measurements. Crit Care Med. 2001;29: 936–939. doi:10.1097/00003246-200105000-00004

19. Kindopp AS, Drover JW, Heyland DK. Capnography confirms correct feeding tube placement in intensive care unit patients. Can J Anesth. 2001;48: 705–710. doi:10.1007/BF03016209

20. Thomas BW, Falcone RE. Confirmation of nasogastric tube placement by colorimetric indicator detection of carbon dioxide: A Preliminary report. J Am Coll Nutr. 1998;17: 195–197. doi:10.1080/07315724.1998.10718746

21. Howes DW, Shelley ES, Pickett W. Colorimetric carbon dioxide detector to determine accidental tracheal feeding tube placement. Can J Anesth. 2005;52: 428–432. doi:10.1007/BF03016289

22. Frakes MA. Measuring end-tidal carbon dioxide: clinical applications and usefulness. Critical care nurse. 2001. pp. 23–26, 28. doi:10.4037/ccn2001.21.5.23

23. Gilbertson HR, Rogers EJ, Ukoumunne OC. Determination of a practical pH cutoff level for reliable confirmation of nasogastric tube placement. J Parenter Enter Nutr. 2011;35: 540–544. doi:10.1177/0148607110383285

24. Metheny N, Reed L, Wierseam L, McSweeney M, Wehrle MA, Ciark J. Effectiveness of ph measurements in predicting feeding tube placement: An update. Nurs Res. 1993;42: 324–331. doi:10.1097/00006199-199311000-00002

25. Robin ED, Whaley RD, Crump CH, Travis DM. Alveolar gas tensions, pulmonary ventilation

and blood pH during physiologic sleep in normal subjects. J Clin Invest. 1958;37: 981–989. doi:10.1172/JCI103694

26. Liu SY, Lee TS, Bongard F. Accuracy of capnography in nonintubated surgical patients. Chest. 1992;102: 1512–1515. doi:10.1378/chest.102.5.1512

27. Ellett MLC. Important Facts About Intestinal Feeding Tube Placement. Gastroenterol Nurs. 2006;29: 112–124. doi:10.1097/00001610-200603000-00004

28. Sayah AJ, Peacock WF, Overton DT. End-tidal CO2 measurement in the detection of esophageal intubation during cardiac arrest. Ann Emerg Med. 1990;19: 857–60. doi:10.1016/s0196-0644(05)81557-4

29. Tutuian R, Castell DO. Gastroesophageal reflux monitoring: pH and impedance. GI Motility online. 2006. doi:10.1038/gimo31

30. Evans DF, Pye G, Bramley R, Clark AG, Dyson TJ, Hardcastle JD. Measurement of gastrointestinal pH profiles in normal ambulant human subjects. Gut. 1988;29: 1035–41. doi:10.1136/gut.29.8.1035

31. Youden WJ. Index for rating diagnostic tests. Cancer. 1950;3: 32–35. doi:10.1002/1097-0142(1950)3:1<32::AID-CNCR2820030106>3.0.CO;2-3

32. Krouwer JS. Cumulative distribution analysis graphs--an alternative to ROC curves. Clin Chem. 1987;33: 2305–2306. doi:10.1093/clinchem/33.12.2305a

33. Coste J, Pouchot J. A grey zone for quantitative diagnostic and screening tests. Int J Epidemiol. 2003;32: 304–313. doi:10.1093/ije/dyg054

34. Elpern EH, Killeen K, Talla E, Perez G, Gurka D. Capnometry and Air Insufflation for Assessing Initial Placement of Gastric Tubes. Am J Crit Care. 2007;16: 544–549. doi:10.4037/ajcc2007.16.6.544

Figure 1

Click here to access/download;Figure;Figure 1.jpg

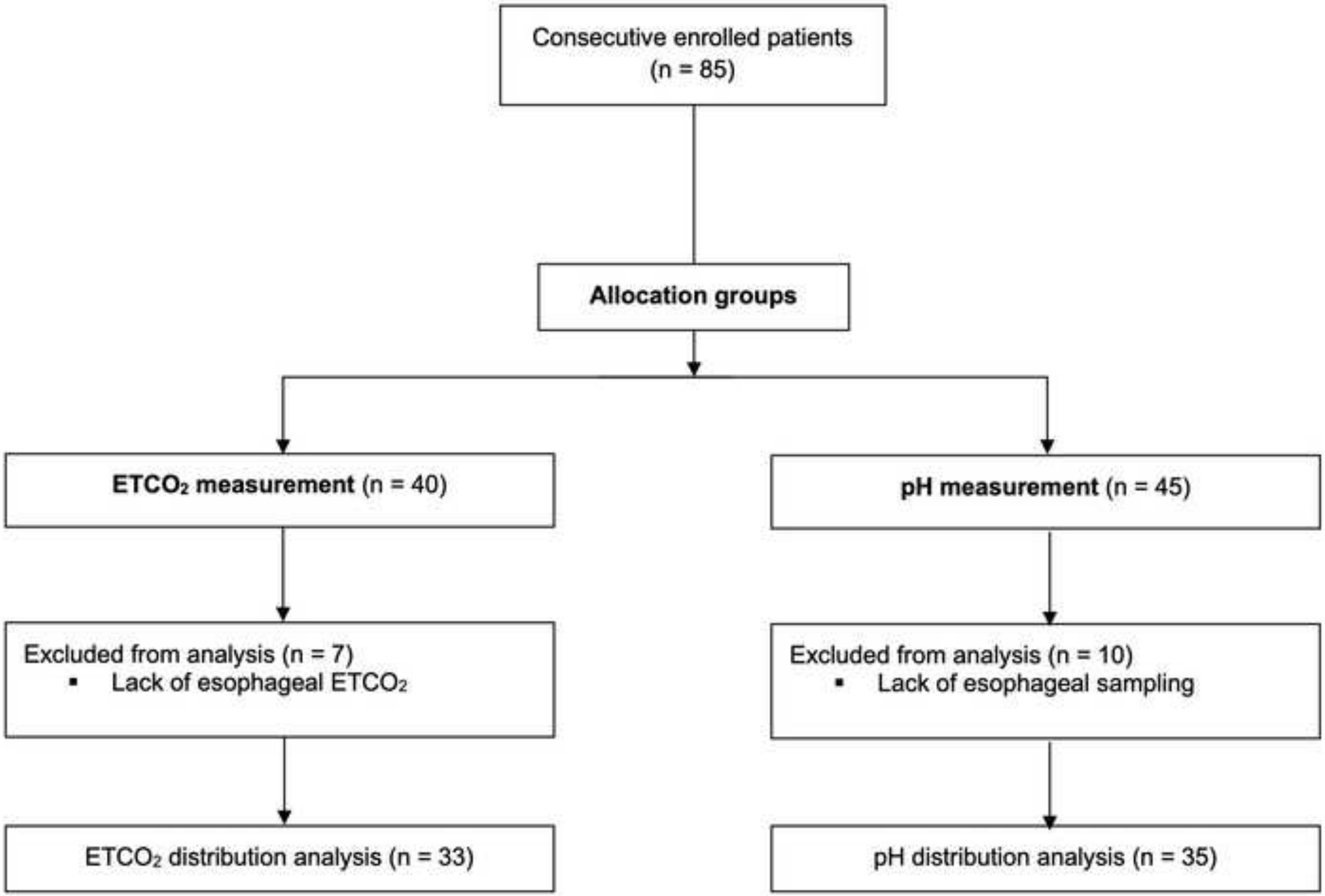

Figure 2

Click here to access/download;Figure;Figure 2.jpg

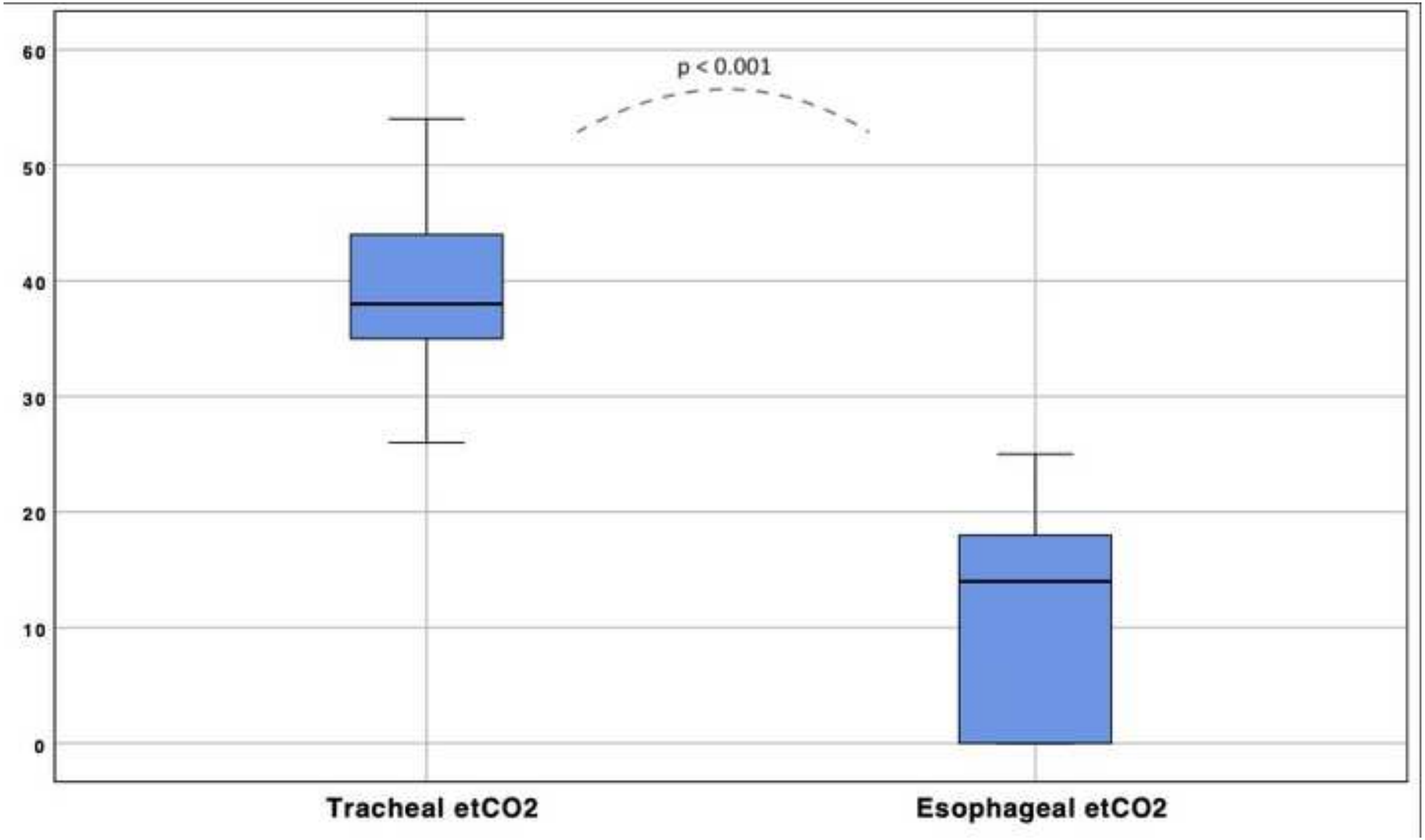

Figure 3

Click here to access/download;Figure;Figure 3.jpg

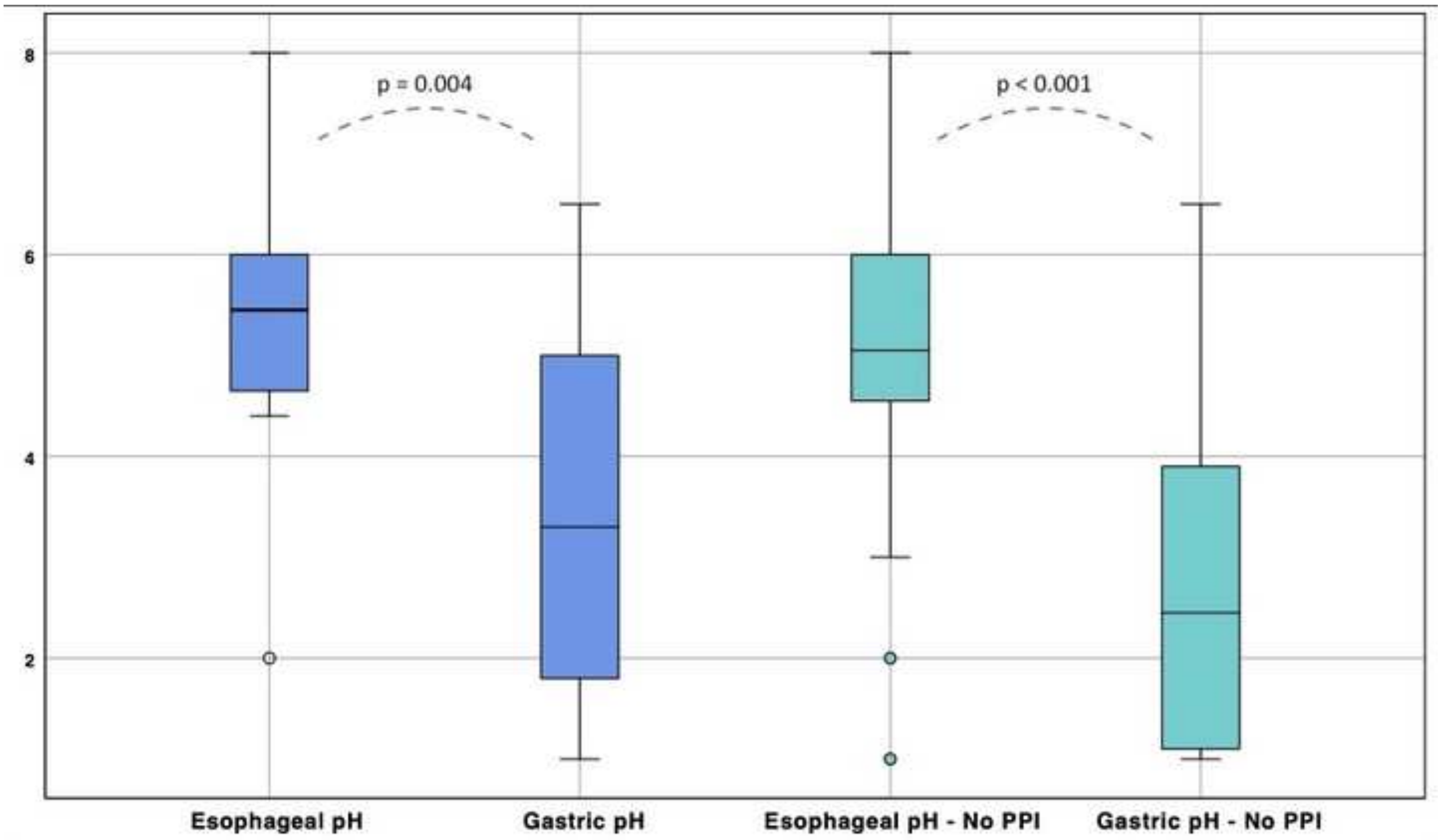

Click here to access/download;Figure;Figure 4.jpeg

the ROC curve of et-CO2 should be better highlighted; it fades now within the Y axis and top horizontalsed

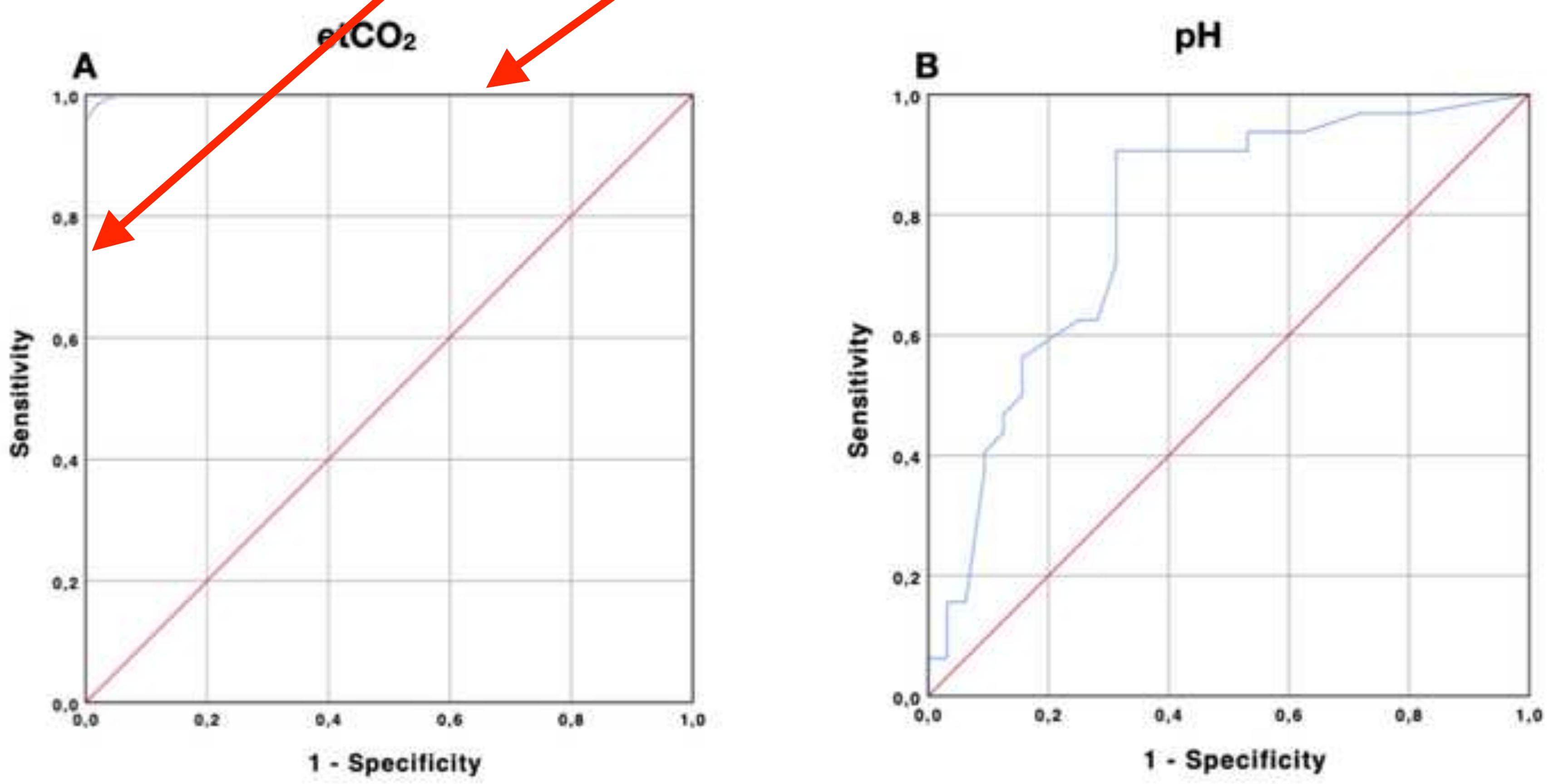

Figure 5

Click here to access/download;Figure;Figure 5.jpg

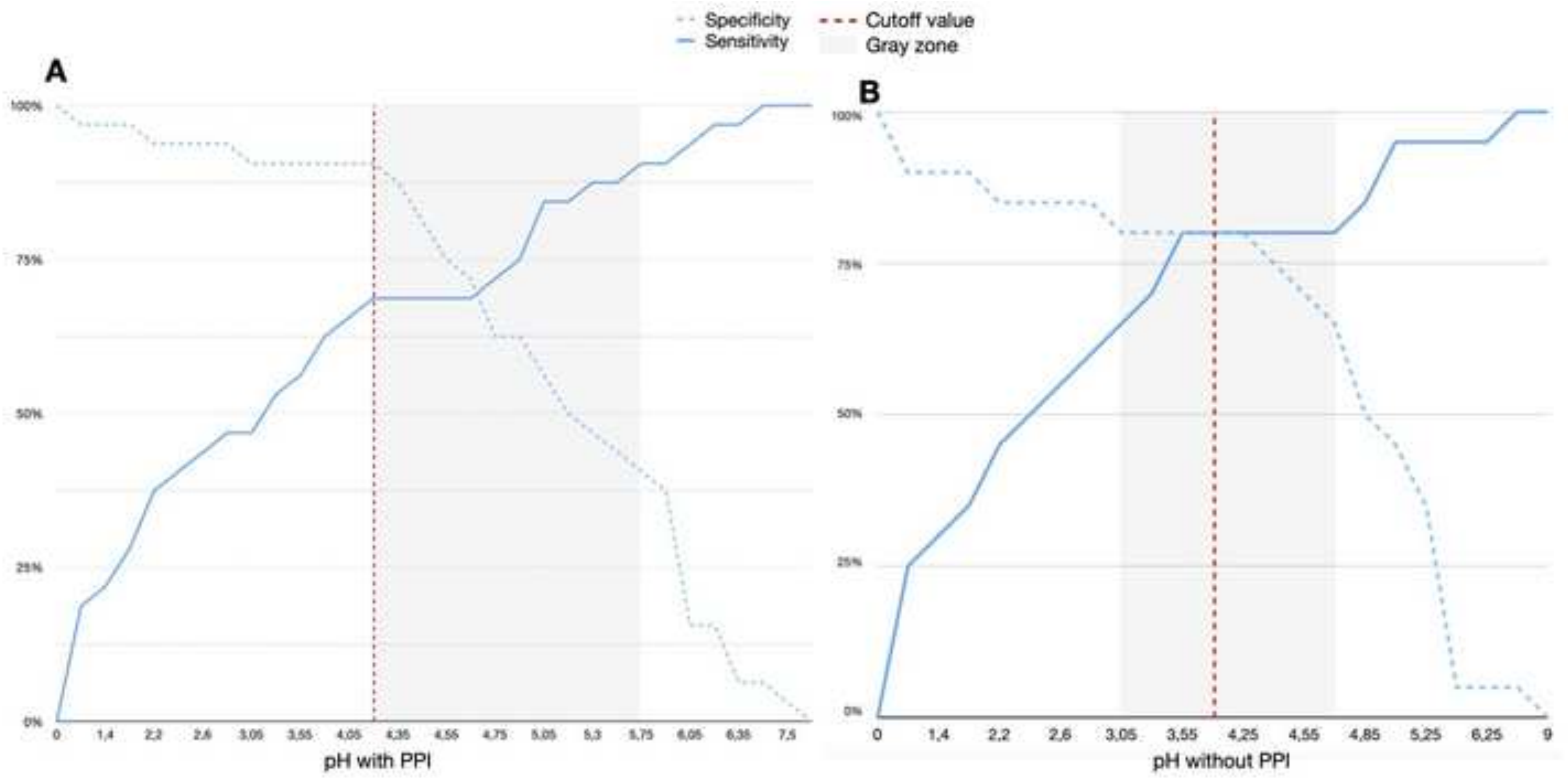

Figure 6

Click here to access/download;Figure;Figure 6.png

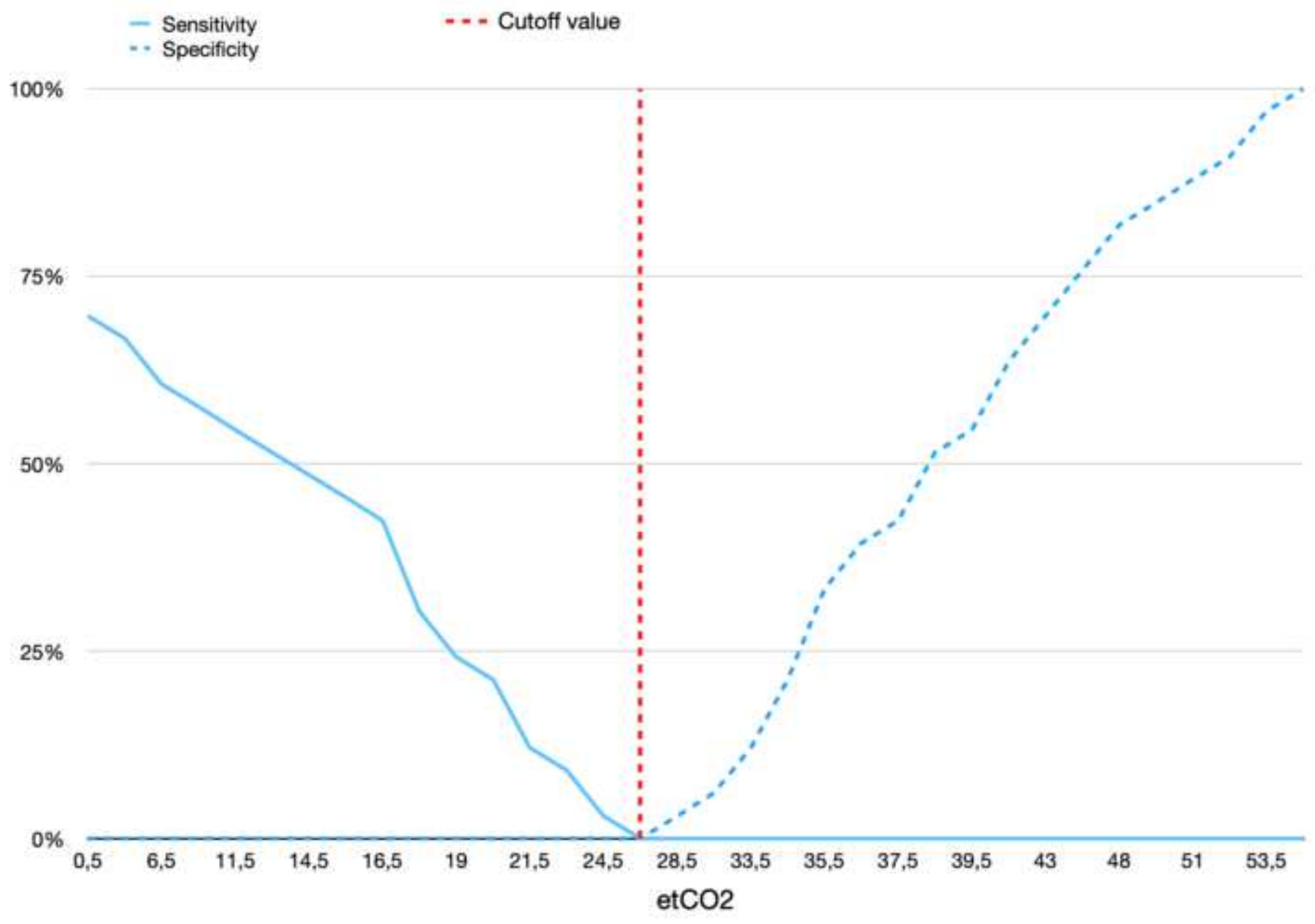

Click here to access/download
**Supporting Information**
ClinicalProtocol_etCO2_gastric_pH_v3_11022020.pdf

Supporting Information

Click here to access/download
**Supporting Information**
CONSORT 2010_Ceruti.doc

Supporting Information

Click here to access/download
**Supporting Information**
Ceruti_MedRxiv.pdf
