# Supplementary material for: Nasogastric tube in mechanical ventilated patients: ETCO2 and pH measuring to confirm correct placement. A pilot study
Source: PLoS One. 2022 Jun 2;17(6):e0269024. doi: 10.1371/journal.pone.0269024 (PMC9162373; doi:10.1371/journal.pone.0269024)
Supplement: Attachment — Submitted filename: review NG paper PR.docx [file pone.0269024.s004.docx]

MAJOR REMARKS:

- Relevant topic and objective, good ideas, nice graphics
- But English language is not OK and is compromising the understanding of the article and study by the reader. English language needs to be reviewed both grammatically as with respect of appropriate use in the context of the study. Evident errors or improper word use are highlighted in yellow in an edited PDF version. From time to time I inserted suggestions in textboxes with red characters.
- The study design could have been much better. There are 2 major weaknesses that could easily have been addressed by a more careful study design.
  1. why were both measurements (EtCO2 and pH) not performed in all patients and measurements analysed separately; this would have allowed to directly compare both methods.
  2. Why was Chest Xray not described and used as a gold standard to be compared with the results of EtCO2 and pH? Are chest X rays available? if sufficiently available why not incorporate in these in an additional analysis and revised article?; I leave it to the editor to decide if such would be advised.
- Abstract reflects insufficiently the study; conclusions in abstract not coherent with methods and study results
- It looks to me as if the use of combined measurement (positive/negative prediction) is a late and awkward interpretation of the results and could have been addressed in a prospective (intentional) way. The advantage of the combined use of both methods is not supported by the way the study is done and described. Furthermore taking the absolute discriminative power of EtCO2 into account the advantage of adding a second method (pH practically inferior) is not clear to me and should be better clarified.
- The EtCO2 method has been recently described in a publication and may therefore have escaped the attention of the writers. This should be reference and discussed in a revised version.

Therefore I recommend thorough revision of the paper.

**Remarks from the reviewer.**

- I have insufficient statistical expertise and did not check the power calculations or the statistic methodology.
- I did not check the supporting additional information.

**Abstract**: inappropriate Discussion; the claim in line 44 “*A device capable of combining the presence of a negative marker with a positive marker could be accurate enough in identifying the correct NGTs positioning*” is neither supported by the methods nor from the results as stated in the abstract.

Line 70: Change to “…. determined: FURTHERMORE the 2 measurements have never been ~~associated~~ COMBINED,….)” REMARK: neither were they combined simultaneously in this study

**Study Design**:

Remarks

Line 81; statement about informed consent

Line 85: please authors should specify explicitly whether there was a clear indication for NG insertion. If this is not the case this suggests that NG tube was inserted for the sake of the study. I reckon the EC has taken this in account.

Line 93; sentence “*Patients in whom pH and/or ETCO2 values were not measurable for technical reasons were excluded and considered as drop-outs.* ” should go to statistical chapter

Line 95-103: What does 1 to 1 randomisation exactly mean; in my opinion this is not coherent with tne rest of this paragraph 96-103. Should be reformulated or clarified (consider using a table…

Line 109 & 116:

- The technique used for NG insertion should be detailed (chin lift, help of an assistant, laryngoscopic guidance; which laryngoscope, finger insertion, Mc Cormack classification ….)
- “*Standard*” NG insertion (line 109) or “*according to local protocols*” (line 116) is not necessarily the same; please rephrase or explain …
- Specify how ventilation was done before intubation; in particular specify if hyperventilation was avoided or not…

Line 123: consider replacing “*esophageal and gastric pH*” by “*samples taken at 20 and 40 cm*”

Line 126 & 127: specify range of EtCO2 values in stead of “around”;

- were values established in the study protocol?; consider elaborating on this in the discussion
- References should be given to underpin these values

Line 130-131: specify range of pH values in stead of “around”;

- were values established in the study protocol?; consider elaborating on this in the discussion
- References should be given to underpin these values

Line 135: “*across the study’s sections*” doesn’t make sense to me; leave it out?

Line 136 & 137: “*by mean and standard deviations (±SDs), or by medians and interquartile ranges (IQR)*” please specify what belongs to pH and what belongs to EtCO2??

Line 138 & 139: “*refusing the null hypothesis of no difference between the two if the p-value was ≤ 0.01*”. I would suggest to phrase the null hypothesis more explicitly.

Line 145-146: description of “grEy zone” needs to be reformulated

Line 147: “*All hypothesis tests*” is this not an over-generalisation ??; does “one tailed” not pertains to distribution and not to all tests

**RESULTS**

Line 153 ”*such as the impossibility to measure pH*”. How often; were there other failure reasons, if so specify. This may be relevant to the study purpose.

**Table 1:**

- PPI intake, Esophageal malformations, Jatal should be stated in both groups; please look this up in group A; likewise COPD - in particular severe emphysema - may be worth while knowing; can this information be added?
- What does esophageal and tracheal value mean, units???
- SD or median to be specified what applies to what
  - SF (min-Max): Min-Max not in the table
  - Likewise Median (25-75^th^); use IQR as an abbreviation: here as well values not in the table
- Consider 2 tables: one for demographics, one for study measurements

Line 163, 164 and 174: Results and statistical should be presented according to groups in stead of using pH or EtCO2 distribution analysis; consider omitting subtitle “distribution analysis”

Line164-174: Please do not reiterate data in table and in results in particular where they are trivial to the study. Prefer table for descriptive and comparative data.

Line 176; enteral feeding or not: should be in patient section

Line 168-169: to be dully rephrased

Line 182-3:legend of Fig 3. “*The subgroup analysis involving patients without PPI showed a greater difference*…”

- Gastric or esophageal pH???? Not clear; again I would prefer elaborating on this in a table
- consider substituting with: “ lower values with greater differences

Line 208: Consider repeating what NND stands for (Numbers nedded to Diagnose

Line 210: likewise for CDA (Cumulative …)

DISCUSSION

Line 230: “when applied together, …”; in my opinion both methods (EtCO2 and pH) were used in the study but the study should not suggest they were combined (applied together) in the same patients!!

Line 259: I suggest using the word “methods” and not “device” as a device is not necessary to combine both methods in a single patient. … both methods are actually readily/easily available in OR/ICU/ER’s, …

Why would a positive marker still be necessary when EtCO2 has a ROC of 1.

The weaknesses of the study designed as I described above should be discussed as shortcomings in particular the lack of using the X rays as a gold standard; this would in my opinion alleviate the need of using pH

With respect to EtCO2 results, these should be discussed in relation to the recently published study from Janita Pak Chun Chau (ref see below).

REFERENCES

- Janita Pak Chun Chau, Xu Liu, Kai Chow Choi, Suzanne Hoi Shan Lo, Simon Kwun Yu Lam, Kam Ming Chan, Jie Zhao, David R. Thompson. Diagnostic accuracy of end-tidal carbon dioxide detection in determining correct placement of nasogastric tube: An updated systematic review with meta-analysis. International Journal of Nursing Studies,Volume 123,2021,104071,ISSN 0020-7489. https://doi.org/10.1016/j.ijnurstu.2021.104071.
