# Supplementary material for: Nasogastric tube in mechanical ventilated patients: ETCO2 and pH measuring to confirm correct placement. A pilot study
Source: PLoS One. 2022 Jun 2;17(6):e0269024. doi: 10.1371/journal.pone.0269024 (PMC9162373; doi:10.1371/journal.pone.0269024)
Supplement: Attachment — Submitted filename: review Nr 2 of Naspgrastric tube positiioning manuscript .docx [file pone.0269024.s006.docx]

Review of

“Nasogastric tube in mechanical ventilated patients: ETCO2 and pH measuring to confirm correct placement. A pilot study..”

Introduction:

- Line 55: Omit “*both*”
- Line 65-66: in the review of litterature when authors say “*the 2 measurements have never been associated*”; authours should consider if it would not be worded better as “*the 2 measurements have never been combined in the same study*”
- Lines 67-69. This sentence suggests this will be the objective of the study which – in my opinion – is not the case (as measurements were not combined but used separately in 2 arms)
- Lines Study 71: The latter (measurements were not combined but used separately in 2 arms) should be explicitly mentioned in the aim of the study.

Methods

- Line 104 – 105. As opposed to Group B, NGT positioning was not verified (by Chest X ray). This should be mentioned.
- Line 115-116: “*Inserted*” to be replaced with “*injected*”;

Results

- Line 185-186 (T-test pH) redundant with Line 180-181; to be improved
- Line 185 replace “*compared TO the whole group*” by “*compared TO the whole group*”
- Line 200: Insert Correct: “*The pH ROC curve analysis for predicting* ***CORRECT*** *gastric placement*”
- Line 211: replace “*The NND obtained for the ETCO2 method* ***resulted*** *1*” with “*The NND obtained for the ETCO2 method* ***was*** *1”*
- Line 211-212: Consider more transparent rephrasing “*The NND obtained for the ETCO2 method* ***was*** *1 while the NND obtained for the pH method was 1” to* “*The NND obtained* ***for misplacement of the NGT*** *with the ETCO2 method was 1 while the NND obtained* ***for correct placement of the NGT*** *the pH method* ***was*** *1.68 (…”*
- *Line 215: “grey’ in stead of “gray”*
- *Line 218:* Should be *“***Fig 5**A” and *“****Fig 5****B” in stead of “4A”* and *“4B”* respectively
- *Has the effect of gastric hernia and reflux been examined in this subset of patients. If YES report. If NOT consider this adding in the discussion section on limitations of the study.*

Discussion

- Line 232-233: *“…. when applied together*”: as this was not the case in this study the authors should be more precise. Furthermore the authors should consider adding an explanation why this was not done (as they explained in their answer to the first review (reviewer #3)
- Line 251: consider replacing “ *… to distinguish between correct positioning in the stomach …*.” With “*… to* ***presume*** *~~distinguish between~~ correct positioning in the stomach …”*.
- Line 278: Consider replacing “*implementation”* by “limitation”
