# Supplementary material for: Nasogastric tube in mechanical ventilated patients: ETCO2 and pH measuring to confirm correct placement. A pilot study
Source: PLoS One. 2022 Jun 2;17(6):e0269024. doi: 10.1371/journal.pone.0269024 (PMC9162373; doi:10.1371/journal.pone.0269024)
Supplement: Attachment — Submitted filename: Response to Reviewers_R2.docx [file pone.0269024.s007.docx]

***Samuele Ceruti, MD***

***Medico Ospedaliero - Servizio di Medicina Intensiva***

*Specialista FMH in Medicina Intensiva*

*Specialista FMH in Medicina Interna*

*Specialista SSMUS in Medicina d'Urgenza*

*Specialista SGUM in Ecografia d’urgenza (POCUS)*

Clinica Luganese SA

Via Moncucco 10 - 6900, Lugano

Tél: +41 (0)91 960.81.08

Nat: +41 (0)76 566.11.81

e-mail: [samuele.ceruti@moncucco.ch](mailto:samuele.ceruti@moncucco.ch)

Emily Chenette, Dr.

Editor-in-Chief

*PLOS ONE*

Lugano, 18^th^ April 2022

Dear Dr. Emily Chenette,

On behalf of my co-authors, I have the pleasure to submit the answers to your reviewers’ comments. We appreciated the reviewers’ suggestions that allow us to clarify our key message. Please note that we have uploaded the two versions of the revised manuscript, one in which the new paragraphs were highlighted and an unmarked version, as required.

**Reviewer#2**

**Thanks for the clarification of the study and manuscript
Just a comment: in my opinion comparison between the 2 groups seems not useful.**

**AND**

**Reviewer #3**

**Introduction:**

**-** **Lines 67-69. This sentence suggests this will be the objective of the study which – in my opinion – is not the case (as measurements were not combined but used separately in 2 arms**

***Response:***

We thank the reviewers for this relevant aspect, appreciating the opportunity to clarify this key-point of the manuscript. Effectively, a comparative and associative analysis between the two methods has not been done yet; in this first preliminary paper, it was necessary to determine the threshold values of ETCO_2_ and pH methods, which has still not reported in peer reviewed paper until today. Once obtained this information, a future paper combining and comparing the two methods will be performed. For this reason, as kindly suggested by the reviewer, we removed indication suggesting any “combination” between two methods, as patients were enrolled in 2 separately arms (lines 65-66, line 73).

**-Lines Study 71: The latter (measurements were not combined but used separately in 2 arms) should be explicitly mentioned in the aim of the study.**

***Response:***

We thank the reviewer for this important aspect. According to previous modifications, we have removed all sentences suggesting this “combining aspect” of the paper, implementing in the text that the ETCO2 and pH measurements were 2 complete separate analysis, without any direct comparations (line 73).

**Methods**

**-Line 104 – 105. As opposed to Group B, NGT positioning was not verified (by Chest X ray). This should be mentioned.**

***Response:***

We thank the reviewer for this suggestion; we have implemented this aspect in the text.

**Results**

**-Line 185-186 (T-test pH) redundant with Line 180-181; to be improved**

***Response:***

We thank the reviewer for this suggestion; we’ve removed the unnecessary text in the figure legend.

**-Has the effect of gastric hernia and reflux been examined in this subset of patients. If YES report. If NOT consider this adding in the discussion section on limitations of the study.**

***Response:***

We appreciate the opportunity to clarify this important aspect of the manuscript; unfortunately, the small group size did not allow us to perform further sub-analysis; for this reason, we have not reported these data. We have reported this aspect in the limit section.

**Discussion**

**-Line 232-233: “…. when applied together”: as this was not the case in this study the authors should be more precise. Furthermore the authors should consider adding an explanation why this was not done (as they explained in their answer to the first review (reviewer #3)**

***Response:***

We thank the reviewer for this important aspect. Currently, a comparative and associative analysis between the two methods has not been done yet; in this first preliminary paper, it was necessary to determine the threshold values of ETCO_2_ and pH methods, still not reported in peer reviewed paper until today. Once obtained this information, a future paper combining and comparing the two methods will be performed. Consequently, we have removed any indication or word suggesting any “combination” between two methods.

**- Grammar corrections into lines 55, 65-66, 115-116, 185, 200, 211, 211-212, 215, 218, 251, 278**

***Response:***

We thank the reviewer for all these suggestions. All grammar proposals were evaluated and modified according to reviewer’s hints.

Finally, we would like to thank the reviewers and the editor for these suggestions, because we feel that the manuscript has improved substantially.

Sincerely,

Samuele Ceruti, MD
